# HBCVTr: an end-to-end transformer with a deep neural network hybrid model for anti-HBV and HCV activity predictor from SMILES

**DOI:** 10.1038/s41598-024-59933-4

**Published:** 2024-04-22

**Authors:** Ittipat Meewan, Jiraporn Panmanee, Nopphon Petchyam, Pichaya Lertvilai

**Affiliations:** 1 Center for Advanced Therapeutics, Institute of Molecular Biosciences, Mahidol University, Nakhon Pathom, 73170 Thailand; 2 Research Center for Neuroscience, Institute of Molecular Biosciences, Mahidol University, Nakhon Pathom, 73170 Thailand; 3 Scripps Institution of Oceanography, University of California San Diego, La Jolla, CA 92037 USA

**Keywords:** HBV, HCV, Transformers, BART, SMILES, Deep learning, Atom-wise tokenization, Fraction-wise tokenization, LBDD, SBDD, Computational biology and bioinformatics, Drug discovery

## Abstract

Hepatitis B and C viruses (HBV and HCV) are significant causes of chronic liver diseases, with approximately 350 million infections globally. To accelerate the finding of effective treatment options, we introduce HBCVTr, a novel ligand-based drug design (LBDD) method for predicting the inhibitory activity of small molecules against HBV and HCV. HBCVTr employs a hybrid model consisting of double encoders of transformers and a deep neural network to learn the relationship between small molecules’ simplified molecular-input line-entry system (SMILES) and their antiviral activity against HBV or HCV. The prediction accuracy of HBCVTr has surpassed baseline machine learning models and existing methods, with R-squared values of 0.641 and 0.721 for the HBV and HCV test sets, respectively. The trained models were successfully applied to virtual screening against 10 million compounds within 240 h, leading to the discovery of the top novel inhibitor candidates, including IJN04 for HBV and IJN12 and IJN19 for HCV. Molecular docking and dynamics simulations identified IJN04, IJN12, and IJN19 target proteins as the HBV core antigen, HCV NS5B RNA-dependent RNA polymerase, and HCV NS3/4A serine protease, respectively. Overall, HBCVTr offers a new and rapid drug discovery and development screening method targeting HBV and HCV.

## Introduction

Hepatitis B virus (HBV) and Hepatitis C virus (HCV) are two blood-borne viruses that have been causing major global health concerns. Globally, it is estimated that over 296 and 58 million people are chronically infected with HBV and HCV, respectively, with approximately 1.5 million new infections with each virus annually^1–6^. In addition, there are estimated 820,000 and 300,000 deaths from HBV and HCV complications each year^7,8^. Infection of HBV and HCV can cause liver diseases that progressively develop into more serious diseases, such as cirrhosis and hepatocarcinoma, which are major causes of death^9,10^. Even though HBV and HCV possess distinct viral genetic materials, in which HBV contains double-stranded DNA (dsDNA), and HCV contains positive-sense single-stranded RNA (+ssRNA), they infect hepatocytes^11,12^. HBV and HCV share similar transmission routes via blood contact, thus increasing the chance of coinfection. HBV-HCV coinfection highlights the higher risk of viral virulence enhancement, leading to more severe liver diseases and an increased risk of hepatocarcinoma progression compared to a single infection with HBV or HCV^13–16^.

Preventive vaccines are currently available for single infection with HBV. However, there is no vaccine for HCV^17–19^. The antiviral development efforts against HBV mainly target viral proteins essential for viral replication and pathogenesis, such as the viral nucleocapsid hepatitis B core antigen (HBcAg). HBcAg plays a crucial role in the viral assembly^20–22^, the host immune response regulation, and the progression of liver fibrosis and carcinogenesis^23–31^. In contrast, the development of antivirals for HCV focuses mainly on direct-acting antivirals (DAAs) designed to inhibit NS3/4A serine protease and NS5B RNA-dependent RNA polymerase (RdRp), which are crucial for HCV viral protein activation and viral genome replication, respectively^32^. Although the existing small molecules have shown efficacy against single HBV or HCV infections, co-infected patients require consistent monitoring and more potent antivirals for therapy^19^. Therefore, there is a critical and immediate need for more effective antivirals against HBV, HCV, and their coinfection.

Two main types of in silico approaches are available in the drug design process: structure-based drug design (SBDD) and ligand-based drug design (LBDD). The SBDD employs computational chemistry tools to identify the binding pocket, perform virtual screening, and find the candidates. This allows structure optimization of the candidates based on the binding energy between the identified binding site of the three-dimensional target protein and the small molecules^33–35^. However, the main limitation of the SBDD emerges when there is no available accurate structure of the target proteins^36^. Even though protein structure prediction tools such as AlphaFold and RoseTTAFold have been developed, they may not provide sufficiently accurate structures for practical use in SBDD^37–39^. In addition, the protein pocket selection is another challenge in the SBDD method, especially in proteins with multiple domains, since each pocket may contribute to the protein function differently^40,41^. Therefore, using uncharacterized protein structures or binding pockets in SBDD should be approached with caution, as it may lead to a high rate of false positives^42^.

Unlike SBDD, the LBDD approach does not depend on the highly accurate structure of the target proteins, circumventing the limitations of SBDD. The LBDD approach has recently become more prevalent in drug discovery applications due to the availability of big data and high computing power. The principle of the LBDD method utilizes the multi-target quantitative structure–activity relationship (QSAR) and multi-tasking quantitative structure-biological effect relationships (QSBER). These approaches describe the compounds’ biological activities or chemical properties based on their molecular structures^43–47^. These strategies lead to various applications in multi-target or pan-antiviral drug discovery^48–51^. Furthermore, the applications of machine learning (ML) and deep learning (DL) techniques have recently become prevalent in the LBDD framework due to their high capability to learn and integrate the relationship between molecular representations and their biological activities from big data^52–54^. The applications of ML- and DL-based models also facilitate rapid virtual screening processes, enhancing the discovery of novel antiviral compounds^55–58^.

**Figure 1:**
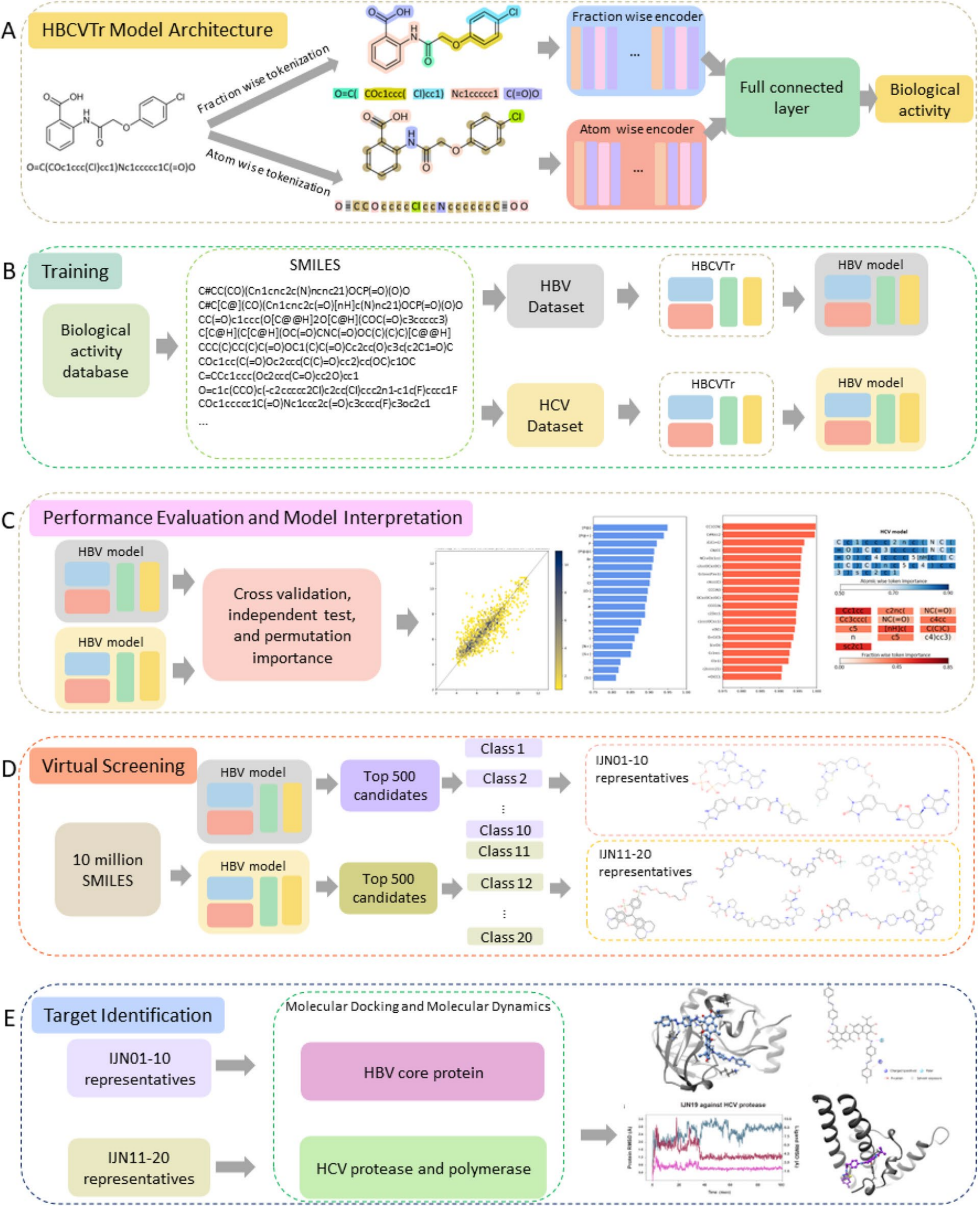
HBCVTr model architecture and proposed drug discovery process outline. (**A**) Illustration of the HBCVTr model structure, featuring atom-wise and fraction-wise encoders and neural network layers. (**B**–**E**) Outlines of the proposed drug discovery process, including training HBCVTr models with HBV and HCV datasets, conducting a virtual screening process using trained models with 10 million compounds, and identifying the targets of representative molecules through molecular docking and molecular dynamics against known HBV and HCV drug targets.

In this study, we introduce HBCVTr, a novel virtual screening tool developed within the framework of the LBDD paradigm (Fig. 1). This approach utilizes a deep learning algorithm to train on a dataset of small molecules represented by a simplified molecular-input line-entry system (SMILES)^59–61^ with known biological activity against HBV and HCV. The SMILES encoding part of the model was built from transformer architecture, which comprises multi-head attention layers. This architecture allows an exceptional capacity to contextualize large datasets by analyzing the interrelationship within sequential data^62,63^. Its versatility and effectiveness have led to widespread applications in various fields, including bioinformatics, cheminformatics, and drug discovery^64–71^. Furthermore, deep neural networks (DNNs) were incorporated into the HBCVTr model to predict the biological activity of small molecules. The utilization of DNNs has demonstrated success in regression tasks across various fields, notably in predicting molecular activities from the molecular structures^72–74^. Here, we employed the end-to-end model that integrates the encoders of the transformer architecture with the DNNs to predict the biological activity of small molecules against HBV and HCV. This prediction is based on tokenized SMILES via atom-wise and fraction-wise tokenization. We applied permutation token importance methods to interpret the model by determining the significance of each molecular substructure contributing to inhibition activities against HBV and HCV. The trained models were benchmarked with other well-known machine learning algorithms and an existing SMILES encoding method. The models were then employed in virtual screening against 10 million small molecules to identify the novel bioactive compounds targeting HBV and HCV. We performed molecular docking and molecular dynamics simulations on the top candidates against selected HBV and HCV target proteins, including HBV HBcAg, HCV NS3/NS4A, and HCV NS5B, to validate our findings. These allow us to assess the binding energy, favorable conformation, and stability between the candidates and their targets.

## Materials and methods

### Collection of SMILES and activity dataset

The antiviral activity assay data of HBV and HCV were retrieved from ChEMBL database (https://www.ebi.ac.uk/chembl/)^75,76^. In total, 7726 and 20,485 compounds have been reported for their experimental results against HBV and HCV, respectively. Due to the variability of biological activity in raw data, only the biological activity data which contain the standard types of IC_50_, EC_50_, Ki, and Kd, the standard relations of ‘ = ’ and the standard units of ‘nM’ or ‘M’ were retained and used for training and testing the model. The activity values were then converted to a comparable standard unit in a negative logarithmic scale. The standard unit was defined as pACT with the formula:

1 $$pACT \, = \, - log10\left( {IC_{50} , \, EC_{50} , \, Ki, \, or \, Kd \, in \, molar \, concentration} \right)$$

where IC_50_, EC_50_, Ki, or Kd are the half-maximal inhibitory concentration, half-maximal effective concentration, inhibitory constant, and dissociation constant, respectively.

All other antiviral activity standard types were excluded due to the inability to interpret them into a single unit, pACT. For SMILES with multiple available pACT values, we retained only the highest pACT, aiming to train our models to predict the highest pACT for each SMILES. After filtering, HBV and HCV’s antiviral activity assay data contain 1941 and 7454 compounds, respectively. Among these compounds, the training, validation, and independent test datasets were randomly split by 72%, 8%, and 20% of the total dataset, respectively, using the Scikit-learn package^77^. For virtual screening, 10 million compounds were compiled from multiple databases, including eMolecules (https://www.emolecules.com/)^78^ and ChEMBL^75,76^.

### SMILES preparation and tokenization

**Figure 2:**
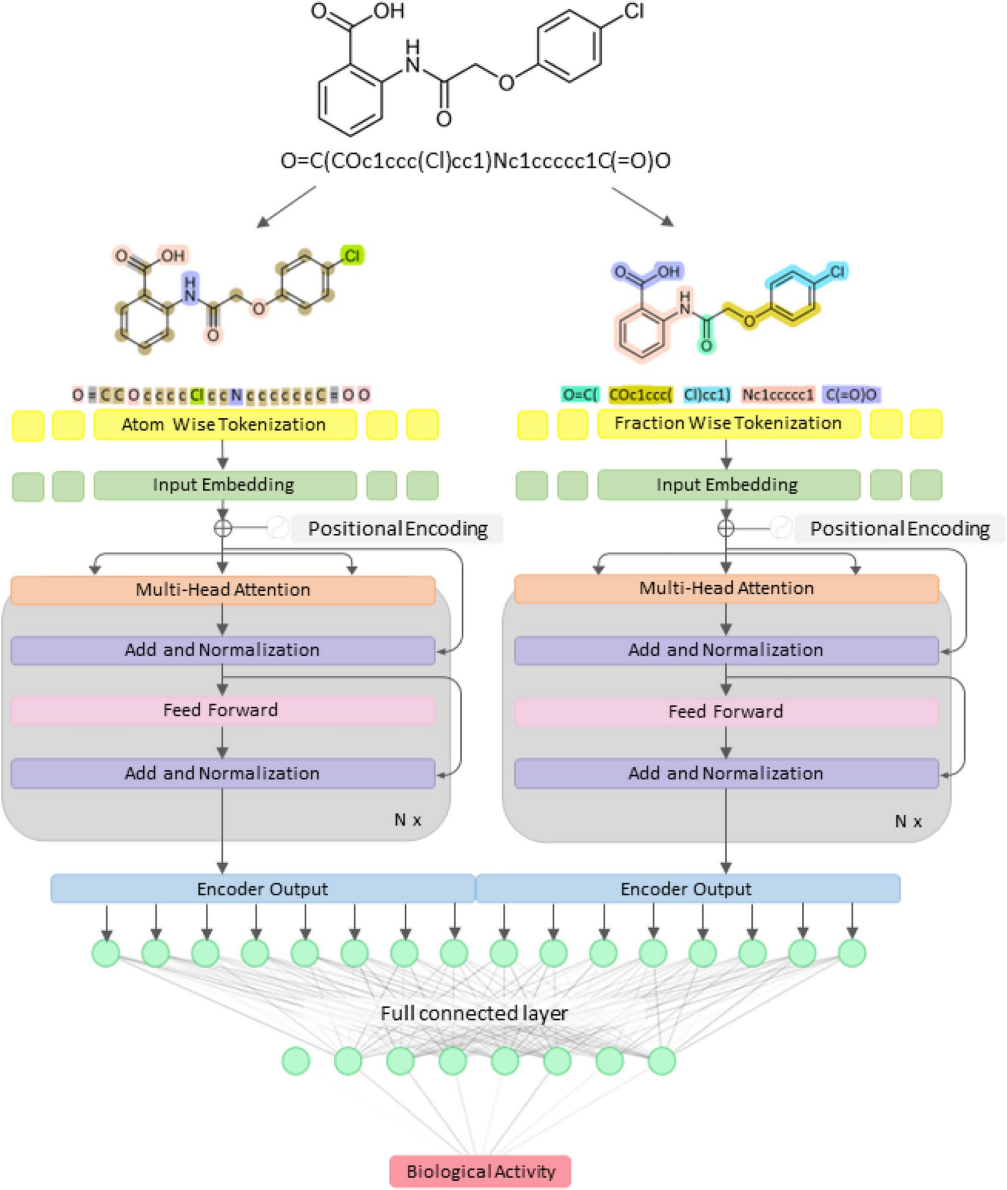
The illustration of the HBCVTr model architecture. Atom-wise and fraction-wise tokenization were applied to the input SMILES chemical structures. These tokenized inputs were processed and passed on to a deep neural network in an end-to-end fashion, yielding the prediction of the biological activity.

The molecular structures of small molecules, represented as SMILES notations^59–61^, were preprocessed to remove salts and converted into canonical SMILES using the RDKIT package (https://www.rdkit.org/)^79^. The canonical SMILES were subsequently dissected into atom-wise and fraction-wise tokens. The atom-wise tokenization technique identified individual atoms and bonds, while the fraction-wise tokenization focused on identifying unique fractions or functional groups within the molecules based on the distinctive pattern of SMILES. Both tokenization techniques were processed using the tokenizer function implemented in SmilesPE (https://pypi.org/project/SmilesPE/)^80^, and DeepSMILES (https://github.com/baoilleach/deepsmiles/)^81^ packages. All preprocessing and tokenization tasks were carried out in Python environment (version 3.8.10)^82^. The total vocabularies from atom-wise and fraction-wise tokenization comprised 274 and 3093 unique tokens, respectively. These sequential tokens were transformed into sequence vectors, representing the index number corresponding to the presence of each token within the original SMILES, for input into the HBCVTr model. All vectors were adjusted to 250 dimensions through truncation or padding. As a result, each molecule was represented by two distinct 250-dimensional vectors, corresponding to atom-wise and fraction-wise tokenization techniques, respectively. Illustrative applications of the atom-wise and fraction-wise tokenization techniques are presented in Fig. 2.

### Model architecture and training

Our novel virtual screening tool methodology, HBCVTr, was fundamentally based on a bidirectional and auto-regressive transformer (BART) architecture, which was adapted for a regression task to predict the biological activity against HBV and HCV using SMILES notations (Fig. 1B)^83,84^. This approach utilized positional encodings to represent the sequential information of tokenized SMILES before inputting into the model. The model contained two encoders: the first encoder processes atom-wise tokenized items, while the second encoder processes fraction-wise tokenized items. These encoders contained multi-head attention layers that learned the contextual relationships of each token in relation to the prediction of biological activity. The outputs from both encoders were concatenated into a single output layer and passed through into fully connected layers designed for biological activity prediction (Fig. 2). Each hidden layer comprised a linear layer followed by the rectified linear unit (ReLU) activation function. The output of the final layer was processed through a regression head, a single linear unit, to output predicted pACT values.

To implement our methodology in predicting biological activity, we separately trained and tested two distinct datasets, HBV and HCV, aiming to predict the biological activity of small molecules against each virus. The training phase involved optimizing learnable weights from both transformers and regression layers based on normalized pACT values. We employed a mean squared error loss function for training. Models that demonstrated minimal loss on the validation sets were saved to ensure robust training and avoid overfitting. The number of learnable weights and hyperparameters were varied to achieve the optimum training procedure. We utilized the grid search for the following hyperparameters: learning rate, number of epochs, dimension of encoder outputs, number of attention heads, number of hidden layers, encoder feed-forward dimension, dropout rate, weight decay, and the number of hidden units and hidden layers in the regression model. Models were built, trained, and tested using transformers (https://pypi.org/project/transformers/)^85^ and Pytorch (https://pytorch.org/)^86^ packages in a Python environment (version 3.8.10). Details on the number of learnable weights and layers, along with other hyperparameters, are shown in Table 1, while search details are provided in Table S1.

**Table 1:** Model configurations and hyperparameters of HBCVTr models.

Model configurations and hyperparameters	HBCVTr	
	Atom-wise encoder	Fraction-wise encoder
Total encoder dimension (d_model)	512	512
Number of attention heads	64	64
Number of encoder layers	6	6
Number of feed-forward dimensions	4096	4096
Vocabulary size	274	3093
Max length	250	250
Learning rate	0.00001	
Weight decay	0.001	
Regression deep neural network size	[1024, 640, 1]	

### Evaluation criteria

To evaluate the models, we utilized a variety of well-known machine learning models for the regression task of predicting pACT values for small molecules targeting HBV and HCV. The selected models included linear regression (LR), k-nearest neighbors (k-NN), support vector regression (SVR), random forests (RF), decision trees (DT), gradient boosting machines (GBM), ridge regression (RR), and adaptive boosting (AdaBoost). These machine learning models predicted the pACT values based on 1024-dimensional output vectors from pre-trained transformers on approximately one million SMILES from the eMolecules database^78^. Additionally, we employed the Mol2vec tool (https://github.com/samoturk/mol2vec)^87^, which converted SMILES into vector representation by identifying molecular substructures. This method was combined with GBM for training and testing in pACT values prediction, providing a benchmark comparison with our methodology. Our comparison also extended to the models that relied solely on atom-wise or fraction-wise tokenization techniques. To assess the predictive efficiency of all models, we employed six metrics for the regression task: mean square error (MSE), mean absolute error (MAE), root mean square error (RMSE), R-squared, Pearson’s correlation coefficient (PCC), and Spearman rank correlation (Spearman). The metrics for these evaluation criteria are described as follows:

2 $$MSE = \frac{1}{n}\mathop \sum \limits_{i = 1}^{n} \left( {Y_{i} - \widehat{{Y_{i} }}} \right)^{2}$$

3 $$RMSE =\sqrt{MSE}$$

4 $$MAE = \frac{1}{n}{\sum }_{i=1}^{n}\left|{Y}_{i}-\widehat{{Y}_{i}}\right|$$

5 $$R{-}squared = 1 - \frac{{\mathop \sum \nolimits_{i = 1}^{n} \left( {Y_{i} - \widehat{{Y_{i} }}} \right)^{2} }}{{\mathop \sum \nolimits_{i = 1}^{n} \left( {Y_{i} - \widehat{{\overline{Y}}}} \right)^{2} }}$$

6 $$PCC = \frac{{\mathop \sum \nolimits_{i = 1}^{n} \left( {Y_{i} - \overline{Y}} \right)\left( {\widehat{{Y_{i} }} - \widehat{{\overline{Y}}}} \right)}}{{\sqrt {\mathop \sum \nolimits_{i = 1}^{n} \left( {Y_{i} - \overline{Y}} \right)^{2} \left( {\widehat{{Y_{i} }} - \widehat{{\overline{Y}}}} \right)^{2} } }}$$

7 $$Spearman = 1 - \frac{{6\mathop \sum \nolimits_{i = 1}^{n} \left( {d_{i} } \right)^{2} }}{{n\left( {n^{2} - 1} \right)}}$$

where $n$ is the number of total compounds, $Y_{i}$ is the actual biological activity value for compound $i$, $\widehat{{Y_{i} }}$ is the predicted biological activity value for compound $i$, $\overline{Y}$ is the mean of actual biological activity values, $\widehat{{\overline{Y}}}$ is the mean of predicted biological activity values, and $d_{i}$ is the difference in ranks between actual and predicted biological activity values for compound $i$.

Lower MSE, RMSE, and MAE and higher R-squared, PCC, and Spearman values indicate the higher predictive performance of the model.

### Model interpretation

To understand the insight of the prediction made by the HBCVTr model, we employed the permutation importance to identify the significance of each token^88^. The importance of each atom-wise and fraction-wise token was calculated based on the increase of prediction errors or the loss of SMILES’s validity after permuting the tokens, compared with the prediction value of original SMILES. We employed MSE to evaluate the importance of the token between permuted SMILES and original SMILES. We define token importance score as:

8 $$Importance\,score = MSE_{perm} - MSE_{orig}$$

where $MSE_{perm}$ and $MSE_{orig}$ represent MSE from permuted SMILES and original SMILES, respectively. Necessary tokens increase the prediction error or result in invalid SMILES when shuffled, while unimportant tokens do not change the prediction errors when shuffled. The importance of each token was investigated separately in HBV and HCV models. The importance score of each token, specifically for HBV and HCV biological activity prediction, was presented as the average score.

### Virtual screening and pharmacokinetic properties prediction

The optimal models, trained explicitly for HBV and HCV for biological activity prediction, were utilized in virtual screening to identify new potential inhibitors for HBV and HCV (Fig. 1D). We screened a library of 10 million compounds, represented as SMILES, against separate models for both viruses. The SMILES used in virtual screening were preprocessed using the same techniques as the training and validation step. The top candidates exhibiting the highest pACT scores from the prediction were then further evaluated for their preferable physicochemical properties as drug candidates for HBV and HCV treatment. Relevant pharmacokinetic properties, including molecular weight, solubility, lipophilicity, the potential to be pan-assay interference compounds, and structural alerts for potentially toxic chemical substructures, were predicted using the SwissADME web tool (http://www.swissadme.ch/)^89–91^. The molecular distances of the top candidates were determined based on their Tanimoto distance between binary fingerprints. For each dataset, the top 500 compounds with the highest pACT values were selected and clustered based on their Tanimoto similarity distances using the average unweighted pair group method with arithmetic mean (UPGMA) to ascertain the distances between classes^92,93^. The cluster distances of 0.6 or lower were clustered into the same class. The individual Tanimoto distances and the cluster distance are described as follows:

9 $$d(A,B)=\frac{{\sum }_{i=1}^{n}[{x}_{jA}-{x}_{jB}]}{{ {\sum }_{j=1}^{n}({x}_{jA})}^{2}+{ {\sum }_{j=1}^{n}({x}_{jB})}^{2}+{\sum }_{j=1}^{n}({x}_{jA}{x}_{jB})}$$

10 $$d\left( {AB,M} \right) = \frac{1}{2}d\left( {A,M} \right) + d\left( {A,M} \right)$$

where $d(A,B)$ is the Tanimoto distance between binary fingerprints of molecules A and B, ${x}_{jA}$ represents the individual element in molecule A, ${x}_{jB}$ is the individual element in molecule B, and $d(AB,M)$ is the average UPGMA between the joined clusters of AB and the new cluster M.

### Molecular docking and molecular dynamics simulation

The top candidates identified from virtual screening and pharmacokinetic properties were subjected to molecular docking and molecular dynamics simulations to determine their possible target and propose their mechanism as HBV and HCV inhibitors (Fig. 1E). The druggable targets for HBV and HCV were selected from the available structures of viral proteins reported as the potential drug targets^94–97^. The HBV inhibitor candidates were evaluated for their binding affinity against HBV core antigen (HBcAg) (PDB ID: 6J10)^98^, while HCV inhibitor candidates were evaluated for their binding affinity against HCV NS3/4A protease (PDB ID: 6UE3)^99^, and NS5B RNA-dependent RNA polymerase (RdRp) (PDB ID: 4KHM)^100^. The structures of all target proteins were obtained from the Protein Data Bank (https://www.rcsb.org/)^101^. The proteins were then prepared by adding hydrogen atoms, removing all non-standard residues, including solvents and existing ligands, and calculating AMBER ff14SB charges using the Dock Prep feature in UCSF Chimera software (version 1.17.3)^102^. The 3D structures of the ligands were optimized using OpenBabel (https://openbabel.org/) for the ligand input^103^. We utilized Autodock Vina (version 4.2.6) and UCSF Chimera suite for molecular docking to prepare, calculate binding energy, and visualize the 3D binding poses^102,104–106^. The 2D poses of the ligands and the target proteins were visualized using the Proteins Plus web server (https://proteins.plus/)^107–110^. The protein–ligand interactions were further validated using Desmond Molecular Dynamics System (version 6.9), D. E. Shaw Research, New York, NY. The simulation employed periodic boundary conditions, with 150 mM Na^+^ and Cl^−^ added to establish equilibrium. The system, with explicit modeling of water molecules using TIP3P and additional ions (Na^+^ or Cl^−^) to neutralize protein charge, underwent a 100 ns simulation at a constant temperature (300 K) and isotropic pressure (1 bar) using the MTK barostat in an NPT ensemble. The stability of the complexes was analyzed based on root-mean-square deviation (RMSD) and contact frequencies. Residue protonation states were determined at pH 7, and trajectory analysis was carried out using the Schrodinger Maestro (version 13.1.137), Schrödinger, LLC, New York, NY, 2022^111^.

## Results and discussion

### Data preparation and analysis

**Figure 3:**
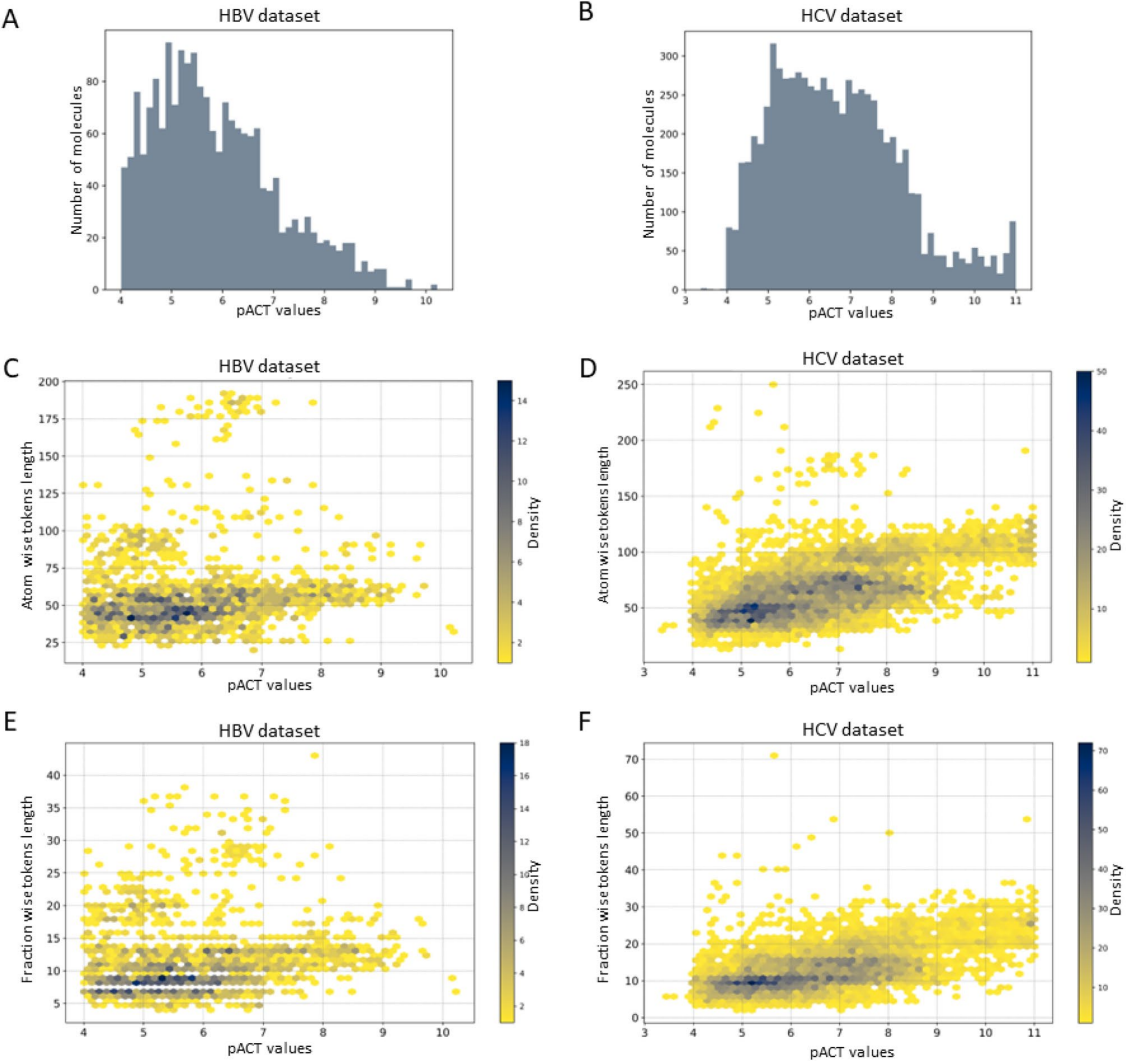
The distribution of pACT values and the length of atom-wise and fraction-wise tokenization of HBV and HCV datasets. (**A**,**B**) Bar plots show the distribution of the number of small molecules against their pACT values for HBV and HCV datasets, respectively. (**C**,**D**) Heat map displays the correlation between the length of atomic tokens and the pACT values for HBV and HCV datasets. (**E**,**F**) Heat map displays the correlation between the length of fraction tokens and the pACT values for HBV and HCV datasets. The color gradient reflects the density of the data in each region.

To evaluate the pACT distribution of the compounds, we extracted, preprocessed, and compiled SMILES and their corresponding pACT values against HBV and HCV into two datasets. The HBV dataset’s pACT values range from 4.01 to 10.22 (60 pM to 100 μM), showing a positively skewed distribution with an average of 5.87 and a standard deviation of 1.21. The HCV dataset’s pACT values range from 3.37 to 11.00 (10 pM to 426 μM), presenting a slightly broader distribution with a mean pACT of 6.67 and a standard deviation of 1.55. The distribution of pACT on HBV and HCV datasets is depicted in Fig. 3A and B, respectively. Analysis of token lengths from both datasets indicates that most entries contain around 25–200 tokens after atom-wise tokenization, while fraction-wise tokenization shows a range of 5–20 tokenized characters. The heatmap depicting the distribution and the size of atom-wise and fraction-wise tokenization objects from both datasets is illustrated in Fig. 3C–F. Therefore, we set a maximum of 250 tokens for both transformer models responsible for atom-wise and fraction-wise tokenization, as this maximum length is expected to cover the majority of entries in the datasets adequately.

### Training and evaluation of the HBCVTr model

The standard BART models utilize the pre-training phase for understanding the context before the specialization through fine-tuning steps, a strategy proven to be productive in studying correlations between the general properties and the SMILES of molecules^83,112^. However, this approach requires extensive libraries and substantial computational resources, and pre-training errors can also carry over to the downstream tasks. For this reason, our methodology avoids the possible pitfalls by skipping the sequence-to-sequence pre-training and proceeding directly to simultaneous training for both the transformer encoders and the DNNs. Nevertheless, a drawback of lacking pre-training is that the encoders do not independently learn the complex grammar or context of SMILES. To address this, we implemented the double transformers employing atom-wise and fraction-wise tokenization methods to identify atoms, bonds, functional groups, scaffolds, and moieties of small molecules. The predictive accuracy of the HBCVTr model, relative to the reference models and the existing SMILES encoding method for biological activity against HBV and HCV, was evaluated using cross-validation and independent tests (Tables 2, 3).

**Table 2:** Cross-validation and independent test results of HBCVTr model on HBV dataset on pACT prediction compared to baseline machine learning and existing models.

Model	Cross-validation					Independent test				
	RMSE	MAE	R-squared	PCC	Spearman	RMSE	MAE	R-squared	PCC	Spearman
LR	0.274	0.212	− 0.911	0.475	0.453	0.263	0.206	− 0.714	0.465	0.466
kNN	0.117	0.095	0.649	0.807	0.740	0.142	0.102	0.497	0.720	0.698
SVR	0.123	0.102	0.614	0.784	0.698	0.138	0.106	0.530	0.731	0.703
RF	0.120	0.097	0.635	0.806	0.728	0.134	0.104	0.555	0.750	0.742
DT	0.181	0.141	0.169	0.592	0.536	0.191	0.140	0.100	0.532	0.533
XGB	0.126	0.102	0.594	0.771	0.696	0.139	0.103	0.518	0.725	0.699
GBM	0.124	0.102	0.606	0.781	0.677	0.143	0.108	0.491	0.703	0.696
Ridge	0.141	0.114	0.492	0.721	0.661	0.169	0.132	0.289	0.645	0.633
AdaBoost	0.143	0.118	0.476	0.731	0.603	0.158	0.132	0.378	0.647	0.603
Mol2vec^87^	0.127	0.101	0.583	0.763	0.697	0.122	0.096	0.625	0.795	0.771
Atom-wise tokenization*	0.117	0.091	0.650	0.807	0.765	0.136	0.102	0.540	0.743	0.717
Fraction-wise tokenization*	0.121	0.092	0.623	0.797	0.757	0.127	0.092	0.598	0.779	0.757
HBCVTr*	0.114	0.090	0.665	0.818	0.757	0.120	0.088	0.641	0.803	0.780

*This work.

**Table 3:** Cross-validation and independent test results of HBCVTr model on HCV dataset on pACT prediction compared to baseline machine learning and existing models.

Model	Cross-validation					Independent test				
	RMSE	MAE	R-squared	PCC	Spearman	RMSE	MAE	R-squared	PCC	Spearman
LR	0.127	0.099	0.622	0.792	0.747	0.135	0.105	0.536	0.734	0.704
kNN	0.108	0.079	0.731	0.857	0.816	0.118	0.085	0.645	0.807	0.784
SVR	0.110	0.085	0.716	0.848	0.812	0.115	0.088	0.660	0.814	0.799
RF	0.106	0.082	0.740	0.863	0.824	0.116	0.088	0.659	0.814	0.792
DT	0.157	0.109	0.428	0.712	0.675	0.158	0.115	0.359	0.666	0.637
XGB	0.107	0.081	0.732	0.856	0.813	0.119	0.090	0.640	0.801	0.779
GBM	0.121	0.097	0.661	0.816	0.764	0.127	0.100	0.587	0.768	0.740
Ridge	0.125	0.096	0.636	0.798	0.751	0.129	0.100	0.574	0.759	0.732
AdaBoost	0.140	0.119	0.543	0.757	0.678	0.145	0.120	0.466	0.693	0.661
Mol2vec^87^	0.116	0.090	0.685	0.830	0.788	0.117	0.091	0.647	0.806	0.784
Atom-wise tokenization^a^	0.101	0.072	0.762	0.874	0.847	0.115	0.084	0.657	0.813	0.796
Fraction-wise tokenization^a^	0.106	0.080	0.737	0.862	0.829	0.105	0.075	0.717	0.847	0.831
HBCVTr^a^	0.097	0.070	0.776	0.881	0.855	0.104	0.074	0.721	0.850	0.831

^a^This work.

Regarding the HBV dataset, the HBCVTr model demonstrated the predictive scores as follows: RMSE of 0.114, MAE of 0.090, R-squared of 0.665, and PCC of 0.818 for cross-validation. The HBV independent test set scores included RMSE of 0.120, MAE of 0.088, R-squared of 0.641, and PCC of 0.803 (Table 2). For the HCV dataset, our model achieved RMSE of 0.097, MAE of 0.070, R-squared of 0.776, and PCC of 0.881 for cross-validation. The HCV independent test set scores were RMSE of 0.104, MAE of 0.074, R-squared of 0.721, and PCC of 0.850 (Table 3). These results, characterized by low RMSE and MAE values alongside high R-squared and PCC values, indicate the potential of the HBCVTr model to exhibit excellent predictive competency against HBV and HCV datasets with minimal error and high accuracy. The performance surpasses other baseline models and the existing SMILES encoding approach, Mol2vec^87^, in cross-validation and independent evaluations.

Furthermore, we utilized Spearman rank correlation to explore the correlation between predicted and actual ranks of compounds in the datasets. Our HBCVTr model achieved Spearman scores of 0.757 and 0.855 for the HBV and HCV datasets, respectively, in cross-validation. The independent test sets scored 0.780 for HBV and 0.831 for HCV, which is higher than the performance of other models. These results indicate that the HBCVTr model effectively predicts the rank of compounds in datasets based on their pACT values. It is an essential capability for predictive models used in virtual screening tasks to identify compounds with high pACT.

Our design and training strategy of the HBCVTr model has demonstrated a significant improvement over models that exclusively rely on either atom-wise or fraction-wise tokenization methods. This enhancement is evidenced by its prediction accuracy on both HBV and HCV datasets in cross-validation and independent tests. The result suggests that incorporating information on atom and connection patterns in SMILES notations through atom-wise and fraction-wise tokenization is crucial for the transformer models to comprehend the contexts and relevant chemical features for predicting biological activity. This approach highlights a successful end-to-end training strategy, where dual transformers and DNNs simultaneously could reduce computational resource and time costs while maintaining learning efficiency. Details of all models used as the reference models for benchmarking are provided in Supplementary Table S2.

**Figure 4:**
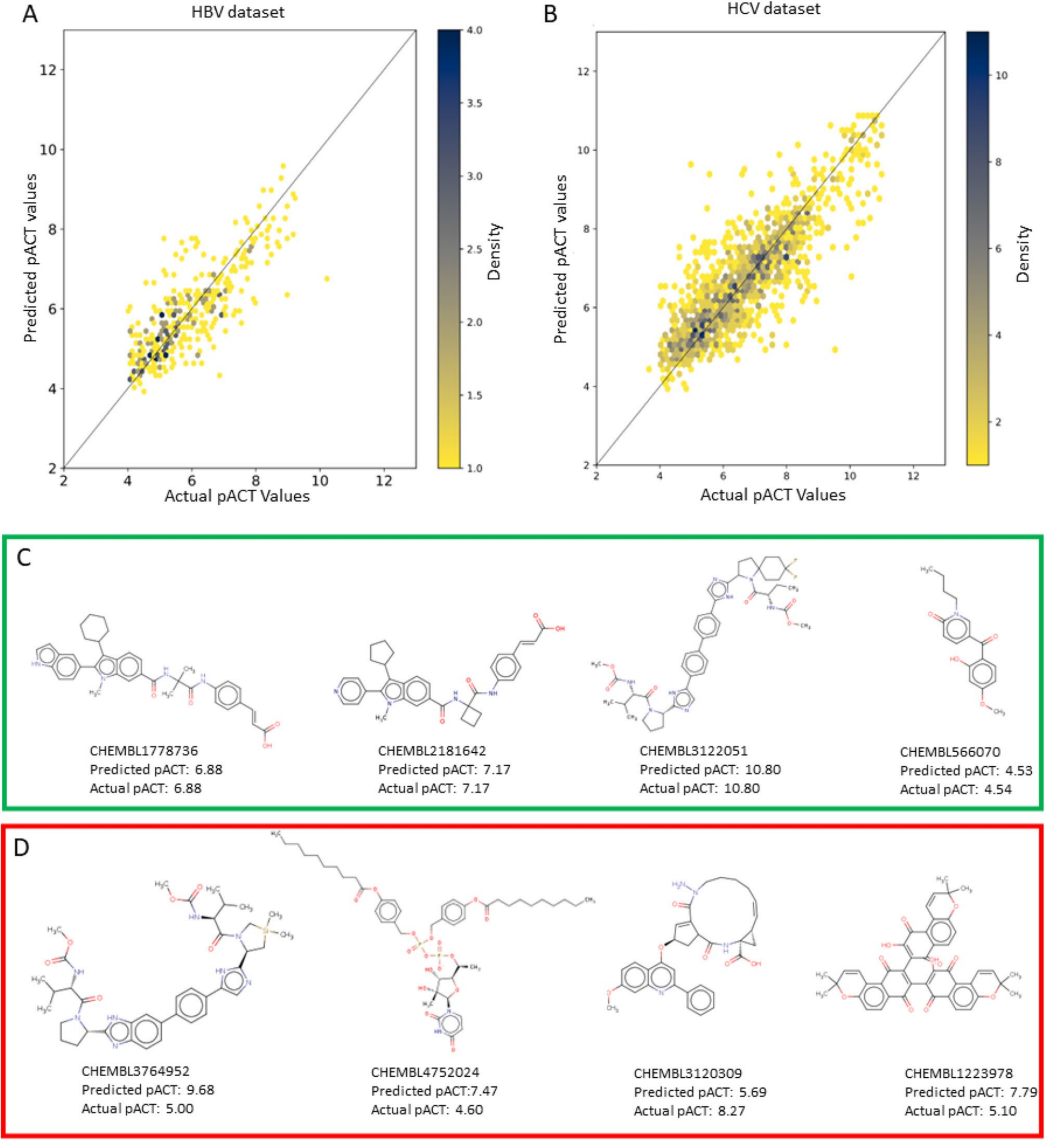
Prediction accuracy and example molecules with predicted and actual pACT values. (**A**,**B**) are heat maps illustrating the correlation between predicted and actual pACT values for the HBV and HCV test datasets, respectively. The y-axis represents the predicted values, while the x-axis represents the actual pACT values. The color gradient shows the data density in each area. Data aligned along the diagonal line indicate high prediction accuracy, whereas data deviating from this line points to prediction errors. (**C**) displays the structures of example small molecules from the HBV and HCV validation datasets outlined in green, which the model predicts with very high accuracy. (**D**) the structures of small molecules from the HBV and HCV validation datasets that exhibit low prediction accuracy for their pACT values are outlined in red.

The heat map plots of the predictive outcomes for the test datasets (Fig. 4A, B) illustrate that the predicted and actual values predominantly cluster along the diagonal, underscoring the models’ highly accurate predictions for most entries in both datasets. However, outliers in the plots indicate occasional over-predictions and under-predictions, suggesting that specific SMILES patterns may challenge the models’ interpretative accuracy. The structures of representative compounds from both datasets and their predicted and actual pACT values are displayed in Fig. 4C, D. Compounds with high prediction accuracy (green in Fig. 4C) typically contain the common fractions, functional groups, and substituents such as indole, imidazole, pyridine, pyrrolidine, benzene, simple aliphatic rings, halogen, ester bonds, and peptide bonds. In contrast, the compounds with low prediction accuracy (red in Fig. 4D) often contain long aliphatic hydrocarbons or complex aromatic systems. The models tend to overpredict the presence of these uncommon fractions in SMILES. Additionally, the molecular fractions with high prediction accuracy are generally smaller compared to those with lower prediction accuracy, which may correlate with poor water solubility, a feature not directly trained in the models. Furthermore, uncommon elements, such as silicon, which is rarely found in small molecule libraries, appear to reduce prediction accuracy. This issue arises from the limited training data available for these elements, leading to models being trained on a small set of examples. This limitation might introduce misunderstanding regarding the impact of these specific elements on biological activity. These issues could be addressed by expanding the size of the training libraries.

**Figure 5:**
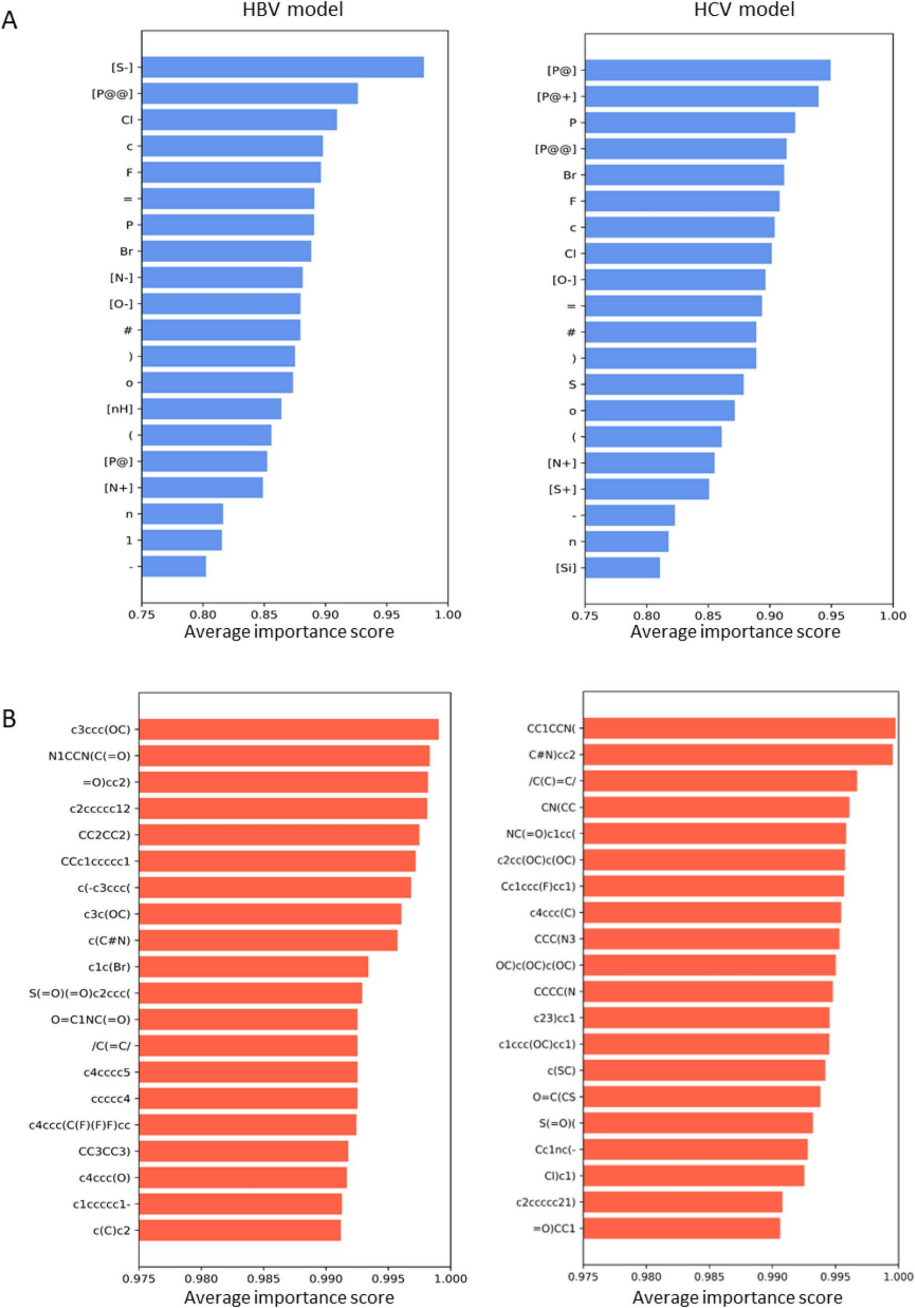
The permutation token importance in the HBCVTr models for the HBV and HCV datasets. (**A**) displays the top 20 most important atomic tokens, and (**B**) shows the top 20 most important fraction tokens. The values depicted represent the relative importance of each token, with higher values indicating greater significance in the models’ prediction.

For the model interpretation, we adopted permutation token importance to investigate the contribution of each atom-wise and fraction-wise token to the prediction pACT against HBV and HCV^88^. Tokens with high scores indicate that the permuted SMILES lost the predictive accuracy or became invalid. The top 20 most crucial atom-wise and fraction-wise tokens from HBCVTr models, pre-trained on HBV and HCV datasets (referred to as HBV and HCV models), are shown in Fig. 5. The results of importance scores from both HBV and HCV models reveal that most impactful tokens span a diverse range of chemical elements and structures. Among these, the top important atom-wise tokens include phosphorus atoms with specific stereochemical configurations, such as [P@], [P@@], and [P@+]; halogens, including fluorine (F) chlorine (Cl), and bromine (Br); heteroatoms, such as phosphorus (P), aromatic nitrogen bonded to hydrogen ([nH]), aromatic oxygen (o), and aromatic nitrogen (n); ions, such as [S^−^], [O^−^], [N^+^], [S^+^], and [N^−^]; types of bonds, such as triple bond (#), double bond ( =), and single bond (–), and truncated tokens, such as ‘(', ')’, and ‘1’. For the fraction-wise tokens, the majority of the tokens with high importance scores in both HBV and HCV models were part of an aromatic system, such as c3ccc(OC), c2ccccc12, CCc1ccccc1, c2cc(OC)c(OC), and Cc1ccc(F)cc1) tokens. These indicate that the models recognize the common and hetero aromatic rings as essential features in HBV and HCV inhibitors, consistent with the previously reported inhibitors^113–117^. In addition, the tokens representing peptide bonds, such as N1CCN(C(= O)), O = C1NC(= O), and NC(= O)c1cc, were found to have high importance scores, suggesting that our models are capable of identifying peptidomimetic compounds, which are frequently used in the development of viral protease inhibitors^118,119^. Furthermore, in the HCV model, high importance scores on amine tokens such as CC1CCN(, CN(CC, CCC(N3, and CCCC(N highlight the significance of nitrogen in alkyl amine or heterocyclic compounds as potential HCV inhibitors^120^. The order of these tokens is crucial, as shuffling them can drastically change the context of the SMILES, leading to a significant loss in prediction accuracy.

**Figure 6:**
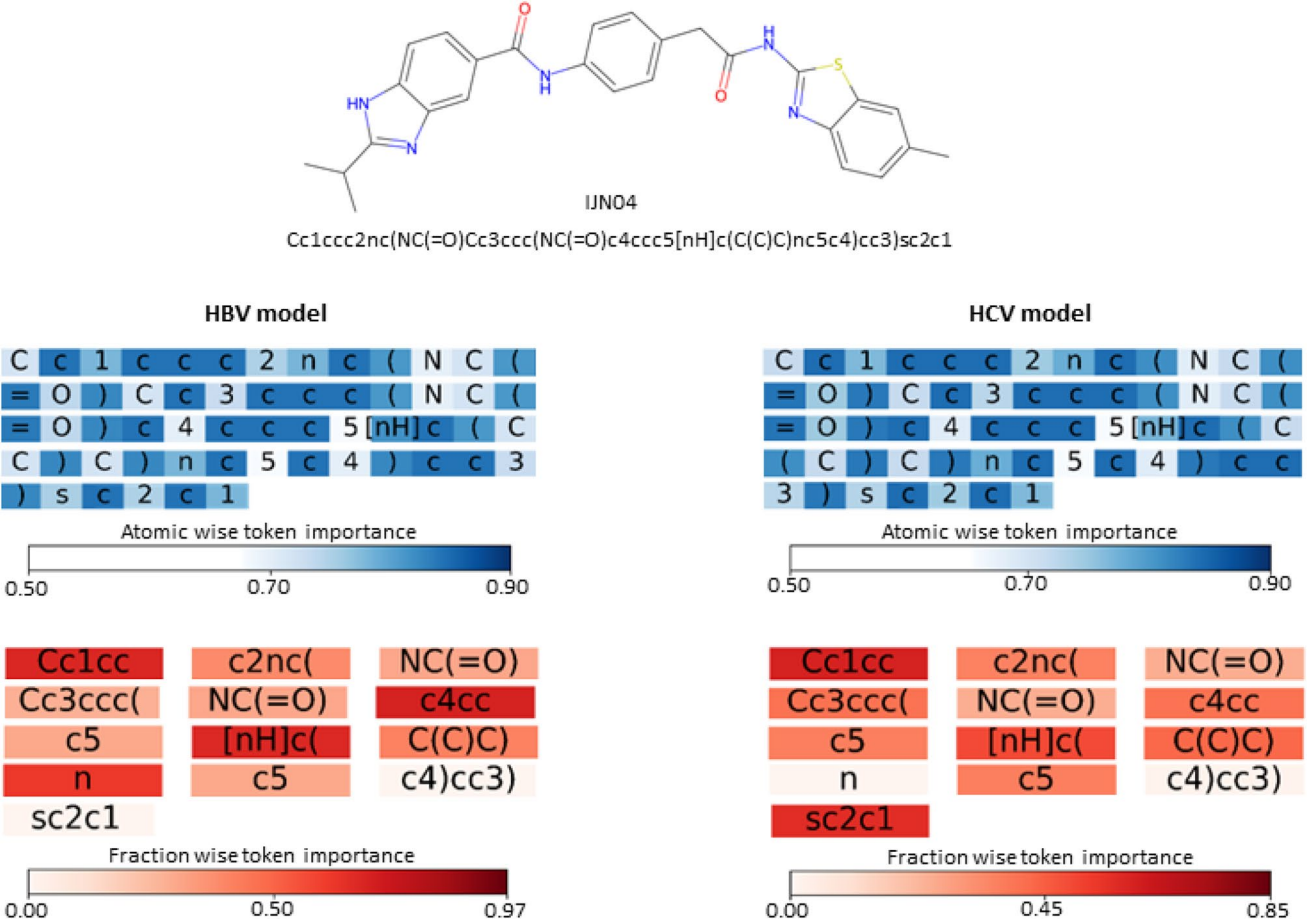
Visualization of the degree of importance of each atom-wise and fraction-wise token based on the interpretation of the HBV and HCV models. The top panel displays the 2D chemical structure and corresponding SMILES representation of IJN04. The middle panel illustrates the degree of importance for atom-wise tokens, indicated in shades of blue. The bottom panel demonstrates the degree of importance of fraction-wise tokens, as indicated in shades of red. The darker shades signify the greater importance of the specific token for biological activity prediction tasks.

Figure 6 presents the tokenized SMILES of the molecule IJN04, processed through atom-wise and fraction-wise tokenization methods. Each token is colored based on its importance score, with darker shades indicating higher importance. Atom-wise tokenization heatmaps for both HBV and HCV models are nearly identical, suggesting the uniform contribution of each atom-wise token in the models’ predictive ability. In contrast, the fraction-wise tokens vary in their contribution to the compounds’ potential as HBV or HCV inhibitors. The HBV model emphasizes the benzimidazole group, with tokens representing this structure, such as c4ccc, [nHc(, and n, assigned high importance scores. Benzothiazole appeared more crucial for the HCV model, with tokens such as Cc1cc and sc2c1 marked highly significant. Utilizing the permutation token importance technique effectively identifies crucial functional groups in HBV and HCV inhibitors. The encoders of HBCVTr models featuring multi-head attentions have demonstrated high proficiency in recognizing these essential substructures, offering valuable insights for developing antivirals targeting HBV and HCV based on specific structural features.

### Virtual screening

Virtual screening is essential for identifying the potential drug candidates in the SBDD approach, wherein target proteins are screened against millions of small molecules in the libraries using molecular docking and molecular dynamics simulation. These processes typically require tremendous computational resources, time, and a high-resolution 3-dimensional structure of target proteins and their identified binding pockets^41^. To circumvent these issues, we employed the virtual screening process using the pre-trained HBCVTr models. Screening 10 million compounds for biological activities against HBV and HCV models can be completed in only 240 h using an NVIDIA 80 GB GPU. Performing molecular docking with AutoDock Vina on the same system would take over 7600 h to process all 10 million compounds^104–106,121^, which is ~ 32 times slower than using the HBCVTr models. This improvement highlights our approach's substantial reduction in computational time and resources.

We obtained two separate datasets of predicted biological activities from HBV and HCV models from each virtual screening. The top 500 compounds from each dataset, ranked by their predicted pACT, were clustered based on molecular similarity into 20 unique classes (10 each for HBV and HCV). The molecules with valid structure and best-predicted pACT values in their class were chosen as representatives, yielding 20 representatives: 10 for HBV, coded as IJN01 to IJN10, and 10 for HCV, coded as IJN11 to IJN20. The structures of all 20 representatives are shown in supplementary Figs. S1 and S2. Tables 4, 5 present the SMILES, ID codes, biological activity predictions, and relevant PK/PD properties for each representative of the major classes from HBV and HCV datasets, respectively.

**Table 4:** PK and PD properties of the representative compounds. SMILES, predicted biological activity against HBV, pharmacological parameters, and docking score are demonstrated for the representatives from the top 10 classes obtained through virtual screening against 10 million small molecules.

Compound ID	SMILES	Predicted pACT	Predicted EC_50_ (nM)	Molecular weight	Predicted pharmacological properties^a^				Docking score (kcal/mol)
					Lipophobicity (logP)^b^	Solubility (logS)^c^	PAINS^d^	Structural alert^e^	HBV core
IJN01	C[C@H](Cn1cnc2c(N)ncnc21)OCP(=O)(O)OP(=O)(O)CO[C@H](C)Cn1cnc2c(N)ncnc21	8.13	7.53	556.41	0.78	− 2.13	0	1	− 5.8
IJN02	Cn1c(=O)n(C)c2cc(CCC(=O)N[C@H]3CCC[C@H](n4cnc5c(N)ncnc54)[C@H]3O)ccc21	7.98	10.57	464.52	2.57	− 2.80	0	0	− 8.2
IJN03	CCCCC(NC(=O)CC(c1ccc(C(C)C)cc1)C(C)C)C(=O)Nc1ccc(C(=O)OC)cc1	7.85	14.19	480.64	4.23	− 7.82	0	0	− 7.0
IJN04	Cc1ccc2nc(NC(=O)Cc3ccc(NC(=O)c4ccc5[nH]c(C(C)C)nc5c4)cc3)sc2c1	7.83	15.01	483.58	2.93	− 7.85	0	0	− 8.8
IJN05	Cc1cc(=O)n2nc(C(=O)NC34CC5CC(CC(O)(C5)C3)C4)nc2[nH]1	7.80	16.12	343.38	1.97	− 3.27	0	0	− 7.1
IJN06	Cc1cc(C(=O)N2CCOC(Cn3nnc4c(N5CCCC5)ncnc43)C2)c(C)o1	7.50	32.12	411.46	3.63	− 3.07	0	0	− 7.4
IJN07	CCc1nn(-c2ccc(C)cc2)c2c1C(c1ccc(N(CC)CC)cc1)C(C(=O)N(CCN)C(C) =O)=C(N)O2	7.77	16.94	530.66	3.88	− 6.45	0	0	− 7.2
IJN08	Cc1ccc(OCC(=O)N2CCN(Cc3cc(=O)n4nc(−c5ccc(F)cc5)sc4n3)CC2)cc1C	7.68	21.22	507.58	4.25	− 5.49	0	0	− 8.4
IJN09	CCC(=O)NCC(c1cccs1)N(CC)CC	7.87	13.46	254.39	2.98	− 2.93	0	0	− 4.7
IJN10	Nc1ccn([C@@H]2CC(O)[C@H](CO)O2)c(=O)n1	7.85	14.16	227.22	0.54	− 0.04	0	0	− 4.7

^a^The relevant pharmacological properties were calculated through the chemical properties prediction function feature implemented in SwissADME^89–91^.

^b^Lipophilicity (LogP) represents the logarithm ratio of a compound’s concentration between octanol and water.

^c^Solubility (logS) represents the logarithms of the compounds’s water solubility in molar concentration (M).

^d^PAINS represents the pan assay interference compound. A high value suggests a high probability that the substance is a PAINS.

^e^Structural alert represents the potential toxicity of the compounds based on the presence of known toxic substructures in the compounds, and a high value suggests a high probability of being a toxic compound.

**Table 5:** PK and PD properties of the representative compounds. SMILES, predicted biological activity against HCV, pharmacological parameters, and docking score against HCV NS3/4A protease and polymerase are illustrated for the representatives from the top 10 classes obtained from virtual screening against 10 million small molecules.

Compound ID	SMILES	Predicted pACT	Predicted EC_50_ (nM)	Molecular weight	Predicted pharmacological properties^a^				Docking score (kcal/mol)	
					Lipophobicity molLogP^b^	Solubility molLogS^c^	molPAINS^d^	Structural alert^e^	HCV NS3/4A protease	HCV polymerase
IJN11	COC(=O)NC(C(=O)N1CCC[C@H]1c1ncc(− c2ccc(− c3ccc4cc(− c5cnc([C@@H]6CCCN6C(=O)[C@@H](NC(=O)OC)C(C)C)[nH]5)ccc4c3)s2)[nH]1)C(C)C	10.65	0.02	794.96	5.71	− 10.13	0	1	− 8.9	− 8.2
IJN12	CN1C(=O)CCC(N2C(=O)c3cccc(NCCOCCC(=O)N4CCN(c5cccc(− c6cnc7ccc(N8CCC[C@@H]8c8cccc(F)c8)nn67)n5)CC4)c3C2 =O)C1=O	9.24	0.31	828.89	5.91	− 6.83	0	1	− 8.7	− 11.3
IJN13	C=CC(=O)N1CCN(c2ccc(CCC(=O)NCCCCCNC(=O)c3cccc(− c4nc(NC(=O)C5(c6ccc7c(c6)OC(F)(F)O7)CC5)ccc4C)c3)o2)C(=O)C1	9.22	0.61	810.84	5.46	− 8.52	0	1	− 7.8	− 10.6
IJN14	CCN(CC)c1ccc(NC2 =C(c3ccc(− n4c(C)ccc4C)cc3)C(=O)N(Cc3ccccc3OC)C2 =O)c(C)c1	8.53	2.92	562.70	5.28	− 7.96	2	1	− 6.2	− 9.3
IJN15	COCCn1ccc2c(NC(=O)c3cn(C4CC4)c4c(OC)c(F)c(F)cc4c3 =O)cccc21	8.59	2.56	467.46	3.86	− 4.57	0	0	− 6.9	− 7.8
IJN16	[N− ]=[N+]=CCCOCCOCCOCCNS(=O)(=O)c1ccc(C2= c3cc4c5c(c3Oc3c2cc2c6c3CCCN6CCC2)CCC[N+]=5CCC4)c(S(=O)(=O)[O− ])c1	9.10	0.80	805.96	1.16	− 5.64	2	5	− 6.8	− 8.7
IJN17	COCc1nnc2n1N(C(=O)Cc1cc(OC)ccc1OC)C(c1cc(OC)ccc1OC)CS2	8.74	1.83	500.57	3.86	− 4.75	0	0	− 6.6	− 7.3
IJN18	C#Cc1cccc(N2C(=O)c3ccc(Oc4ccc(C(C)(C)c5ccc(Oc6ccc7c(c6)C(=O)N(c6cccc(C#C)c6)C7 =O)cc5)cc4)cc3C2 =O)c1	9.31	0.49	718.75	5.83	− 11.20	0	2	− 9.3	− 9.8
IJN19	Cc1ccc(N=Nc2ccc(NC=C3C(=O)C(O)=C(C(C)C)c4cc(C)c(− c5c(C)cc6c(c5O)C(= CNc5ccc(N= Nc7ccc(C)cc7)cc5)C(=O)C(O)=C6C(C)C)c(O)c43)cc2)cc1	9.30	0.50	904.05	8.19	− 16.43	1	2	− 9.9	− 10.2
IJN20	COC(=O)C1c2cc3c(c(O)c2[C@@H](O[C@@H]2O[C@@H](C)[C@H](OC)[C@@](C)(OC)[C@H]2OC)C[C@]1(C)O)C(=O)c1c(O)cc2c(c1C3 =O)O[C@@H]1O[C@@]2(C)[C@H](O)[C@@H](N(C)C)[C@@H]1O	9.42	0.38	787.80	4.72	− 5.47	1	0	− 6.9	− 9.0

^a^The relevant pharmacological properties were calculated through the chemical properties prediction function feature implemented in SwissADME^89–91^.

^b^Lipophilicity (LogP) represents the logarithm ratio of a compound’s concentration between octanol and water.

^c^Solubility (logS) represents the logarithms of the compounds’s water solubility in molar concentration (M).

^d^PAINS represents the pan assay interference compound. A high value suggests a high probability that the substance is a PAINS.

^e^Structural alert represents the potential toxicity of the compounds based on the presence of known toxic substructures in the compounds, and a high value suggests a high probability of being a toxic compound.

**Figure 7:**
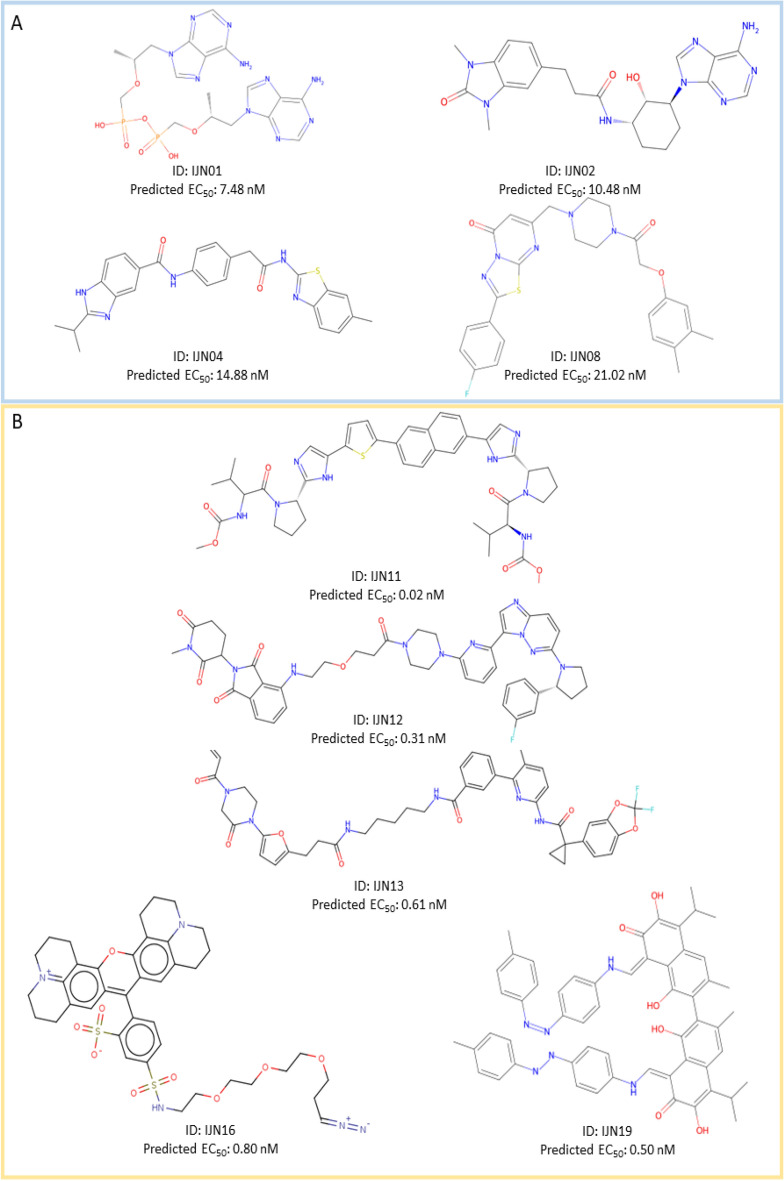
The chemical structures of top representatives and their predicted biological activities against HBV and HCV. (**A**) The 2-dimensional structures and predicted EC_50_ values of the top representative compounds from the HBV dataset include IJN01, IJN02, IJN04 and IJN08, outlined in pale blue. (**B**) The 2-dimensional structures and predicted EC_50_ values of the top representative compounds from the HCV dataset include IJN11, IJN12, IJN13, IJN16 and IJN19, outlined in yellow.

From the analysis of HBV inhibitor candidates, the predicted EC_50_ values in nanomolar range from 7 to 32 nM. Figure 7A displays the structures of the major representatives and the predicted EC_50_ values for the HBV inhibitor candidates. Compound IJN01 possesses adenine and phosphate, critical features in tenofovir disoproxil–an antiviral initially developed to treat human immunodeficiency virus (HIV) and later repurposed against chronic HBV infection^122^. The mechanism of tenofovir disoproxil in inhibiting HBV relies on its cellular conversion from a prodrug to its active form, tenofovir diphosphate, a nucleotide analogue inhibitor against HBV polymerase^123,124^. Similarly to tenofovir, IJN01 and other members of this class could be cleaved at diphosphate moiety, yielding the tenofovir diphosphate, the competitive inhibitor of HBV polymerase. Besides adenine, other heterocyclic compounds, including chromene, pyrazolopyrimidine, thiazolopyrimidine, benzothiazole, isoindoline, benzodioxole, thiazolothiazole, thiazole, oxazole, and substituted benzene, are found among top classes representatives. This finding indicates that these groups are essential features for active HBV inhibitors, aligning with the results of the previous discussion of the permutation token importance. Interestingly, representatives IJN02 to IJN10 have not shown high similarity with the known active HBV inhibitors, highlighting the potential for novel classes of active HBV inhibitors. Regarding the pharmacological properties, most compound classes are within an acceptable range, except for IJN01 and IJN10. These two representatives have slightly low logP values, indicating low cell permeability. In addition, the toxicity prediction for IJN01 suggests slight toxicity due to the presence of Michael acceptor, while the rest of the candidates do not show any toxicity alerts. No pan assay interference structure (PAINS) alerts were observed in these top representatives, suggesting the low likelihood of off-target binding and adverse side effects.

The results of virtual screening using the HCV model show lower EC_50_ than those using the HBV model. The EC_50_ values of all ten representatives range from sub-nanomolar to a few nanomolar levels, specifically from 0.02 to 3 nM. Figure 7B depicts the 2D structures of the representative of major classes among the top candidates for HCV inhibitors alongside their predicted EC_50_ values. Compound IJN11 possesses high similarity to daclatasvir, an effective inhibitor of HCV NS5A, a multifunctional protein crucial for HCV replication^125^. Compound IJN16, the sulforhodamine analogue, commonly used as a fluorescent dye to label DNA/RNA in click chemistry, has shown excellent predicted EC_50_ values^126^. In addition, the model identified the compound IJN20, an analogue of anthracycline antibiotic nogalamycin, as a potent HCV inhibitor^127^. This finding suggests the possibility of repurposing these compounds for HCV treatment. The remaining representatives do not resemble any known HCV inhibitors. The representatives, including IJN12, IJN13, IJN16, and IJN19, tend to have higher molecular weight, implying low water solubility and narrowing the window of concentrations allowable for treatment options. However, the predicted effective doses of the representative compounds against HCV are very low, potentially compensating for their poor cell permeability and water solubility. The compounds IJN14, IJN16, IJN18, and IJN19 were predicted to have a high likelihood of being PAINS compounds, possibly targeting a wide range of off-target proteins. Additionally, these compounds’ structural alert or toxicity prediction is higher than the remaining representatives due to their possession of known toxic and reactive substructures, including diazo, imine, quaternary nitrogen, sulfonic acid, phthalimide, and Michael acceptor. The predicted overall physicochemical properties of most representatives from both the HBV and HCV inhibitor candidates fall within an acceptable range. These predictions indicate that the models can identify favorable atom-wise and fraction-wise tokens and associate them with high pACT or low EC_50_ values. However, since the physicochemical properties were not directly introduced as features to the models during the training process, further investigation of the physicochemical properties of candidates identified through virtual screening using HBCVTr models has proven useful. This additional analysis helps ensure that the top candidates possess desirable PK/PD properties.

### Molecular docking and molecular dynamics simulation of HBV and HCV drug candidates

**Figure 8:**
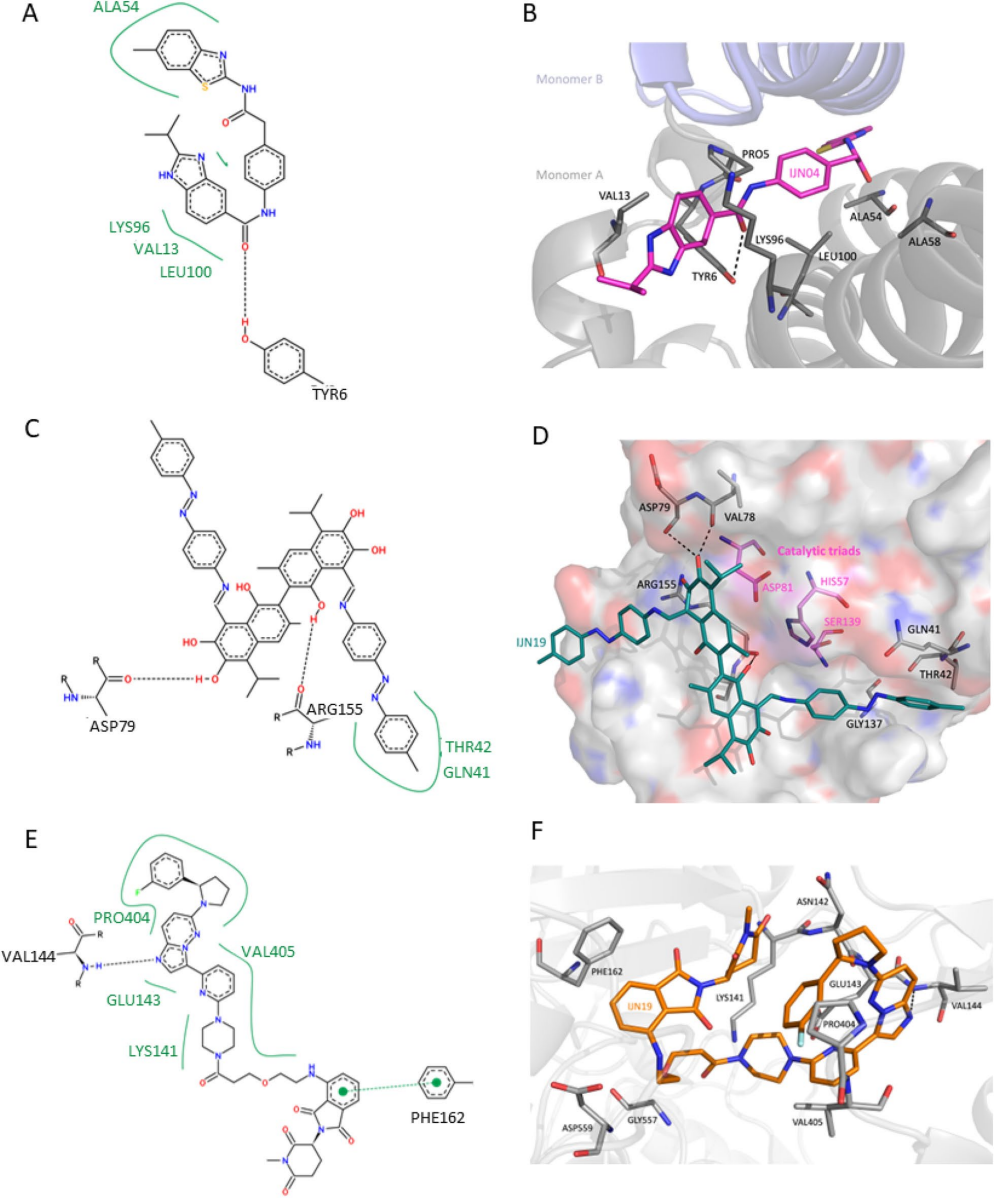
Binding poses and interactions of top representatives against known drug targets of HBV and HCV. (**A**) 2D and (**B**) 3D binding poses of IJN04 against HBV core protein, (**C**) 2D and (**D**) 3D binding poses of IJN19 against HCV protease, (**E**) 2D and (**F**) 3D binding poses of IJN12 against HCV polymerase. The HBV/HCV protein targets are colored in gray, hydrogen bonds are shown as black dotted lines, and the ligands IJN04, IJN19, and IJN12 are shown as magenta, teal, and orange sticks, respectively. IJN compounds and interacting residues are represented as sticks. Oxygen, nitrogen, sulfur, and fluorine atoms are colored red, blue, orange, and green, respectively.

We performed molecular docking of the representative candidates against known target proteins for HBV and HCV using AutoDock Vina^104–106^ to gain insight into the possible binding affinity and inhibitory effects. We selected the HBV core protein (HBcAg) due to its vital function in viral assembly during the viral replication process^128^. Core proteins form a homodimer that further assembles into a trimer of dimers, identified as the nucleating scaffold of viral capsid assembly^129^. The binding energy of IJN01–IJN10 compounds against HBV core protein is shown in Table 4. Among the candidates, IJN04 demonstrated the best binding score of − 8.8 kcal/mol, suggesting a strong binding affinity to the HBV core protein. The 2D and 3D binding poses of IJN04 against the HBV core protein are displayed in Fig. 8A, B, respectively. The docked site of IJN04 was found at the dimer interfaces, with residues including PRO5, TYR6, VAL13, ALA54, ALA58, LYS96, and LEU100 participating in the interactions. The dimerization of the HBV core protein, a crucial event in capsid assembly, can be disrupted by the binding of the small molecules at the interface. This strategy aims to inhibit the structural formation of the virus and, consequently, block the viral replication^98^. Therefore, we postulate that the representative IJN04 may exert its activities on HBV core protein through this mechanism.

**Figure 9:**
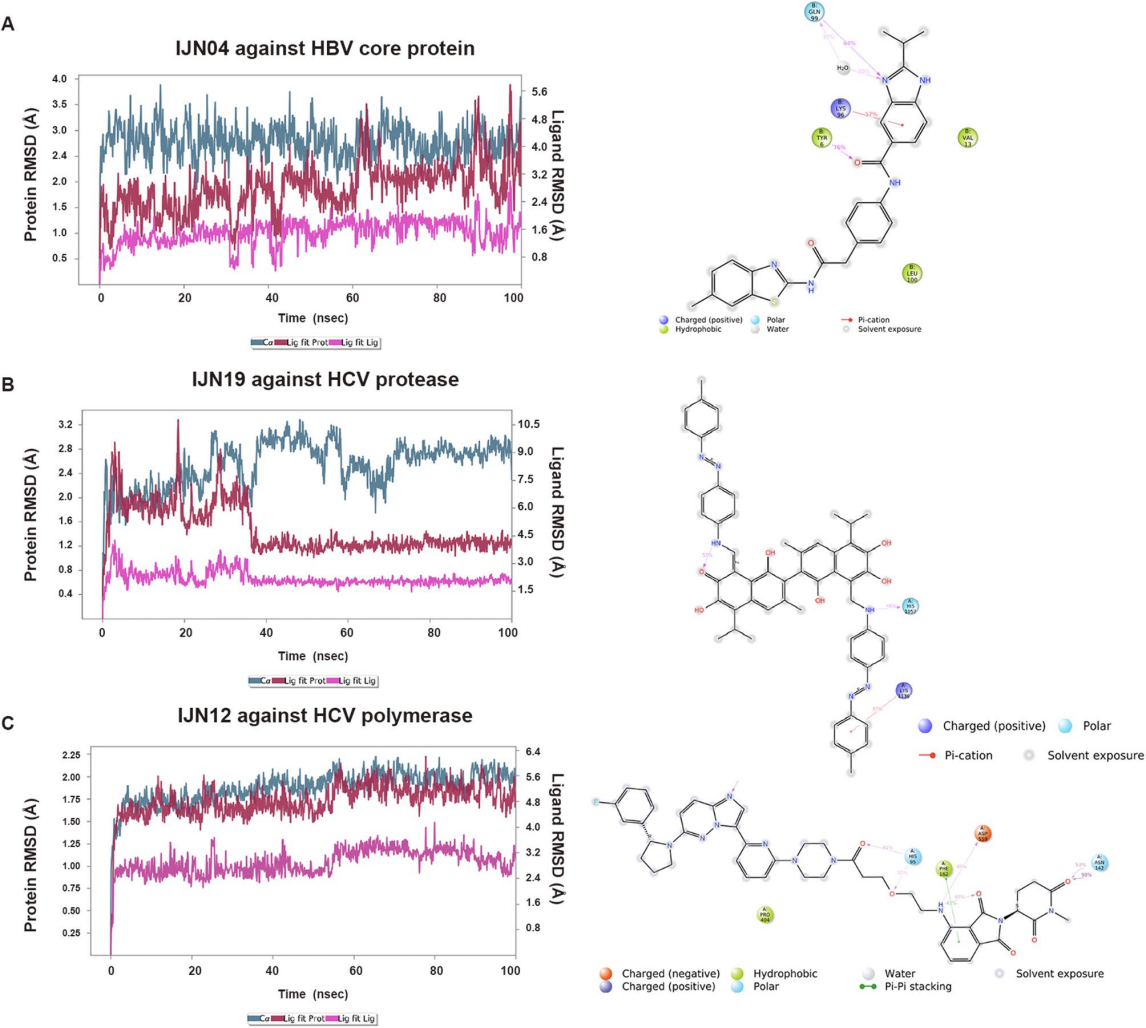
Schematic representation of the root mean square deviation (RMSD) and ligand atom interactions with protein residues over a 100.00 ns simulation. The residue interactions with compounds that occurred for more than 30.0% of the simulation time within the selected trajectory (0.00 through 100.00 ns) are illustrated, providing the most prevalent molecular contacts during the simulation. (**A**–**C**) RMSD plots and detailed ligand atom interactions with protein target residues are presented for (**A**) IJN04 against HBV core protein, (**B**) IJN19 against HCV protease, and (**C**) IJN12 against HCV polymerase.

The docking scores of all ten representatives of HCV inhibitor candidates against HCV NS3/4A protease and NS5B polymerase are shown in Table 5. For HCV NS3/4A proteases, the structure of D168A mutant was chosen for docking because ASP168 is one of the hotspots for resistance-associated substitutions ^130,131^, where this residue contributes to an active-site electrostatic network that modulates the inhibitor binding^99^. The docking pose suggested that IJN19 shows strong binding efficacy against the D168A HCV NS3/4A protease with a binding affinity of − 9.9 kcal/mol. The contact residues within the selected target pocket include GLN41, THR42, VAL78, ASP79, GLY137, ARG155, ALA156, which are located close to the catalytic triads, HIS57, ASP81, and SER139 (Fig. 8C, D). HCV NS3/4A protease recognizes the conserved 3D shape of the peptide substrates rather than conserved amino acid sequences^132^, and the binding mode of IJN19 overlaps with the substrate binding site of NS3/4A at position P1 to P3^132^ (supplementary Fig. S3). The docking result highlights the possibility of IJN19 as a compound to inhibit HCV NS3/4A protease D168A and other variants. In addition, IJN12 exhibited strong binding efficacy against HCV polymerase with a binding affinity of −11.3 kcal/mol (Fig. 8E, F). The contact residues of HCV polymerase include those from the finger domain: LYS141, ASN142, GLU143, VAL144, and PHE162, and from the thumb domain: PRO404, VAL405, GLY557, and ASP559. IJN12 formed the hydrogen bonding through its imidazopyridazine moiety with VAL144 and Pi–Pi stacking interaction between its isoindoline and PHE162, stabilizing the complex. The binding site of IJN12 was found close to the acidic β-hairpin loop and the triphosphate group of NTP, suggesting that IJN12 could inhibit HCV polymerase by interfering with RNA binding. This result highlights that IJN12 may have a strong and specific binding affinity to HCV polymerase. The docking results were subsequently subjected to a 100 ns molecular dynamics simulation. The stability of the complexes formed by IJN04, IJN19, and IJN12 with the respective target proteins, HBV core antigen, HCV NS5B polymerase, and HCV NS3/4A serine protease, was analyzed based on RMSD and contact frequencies. Our findings reveal that IJN04, IJN19, and IJN12 maintained binding to their target proteins throughout the 100 n simulation, as illustrated in Fig. 9.

## Conclusion

In this study, we introduced HBCVTr, a transformers-based model designed for predicting effective drug concentration against HBV and HCV in LBDD applications, utilizing the SMILES notations of small molecules. The models comprised two transformer encoders that capture the provided SMILES’ atomic and fractional information through distinct tokenization levels: atom-wise and fraction-wise. The outputs from these encoders were processed by the DNNs for the regression task of antiviral activity prediction. The models were trained separately in an end-to-end manner using biological activity data from HBV and HCV databases. Our training approach and the model architecture outperformed the traditional training methods, such as pretrain-and-fine-tune strategies, other popular machine learning models, and the existing encoding method in predicting biological activities from SMILES representation. This improvement was evident in both cross-validation and independent test datasets. Specifically, we achieved R-squared values of 0.665 and 0.776 for cross-validation of HBV and HCV datasets, respectively, and R-squared values of 0.641 and 0.721 for the test set of HBV and HCV datasets, respectively. The permutation token impotence analysis identified halogen and heteroatom substitutions, common and hetero aromatic rings, and peptide bonds as essential features for HBV and HCV inhibitors. The pre-trained HBV and HCV models virtually screened 10 million compounds, identified the top compounds that show the highest activities, and classified them based on their structural similarity. Among the novel molecules not yet tested with HBV and HCV, IJN04 emerged as the best representative for the HBV inhibitor candidates, with a predicted EC_50_ of 14.88 nM and favorable binding energy to HBV core protein. IJN12 and IJN19 were identified as top candidates for the HCV’s top representative molecules, with predicted EC_50_ values of 0.31 and 0.50 nM against HCV, respectively. Molecular docking and molecular dynamics simulation further supported the potential of IJN12 and IJN19 as novel viral inhibitors targeting HCV polymerase and protease, respectively. The HBCVTr model’s use of atom-wise and fraction-wise tokenization techniques has exhibited its ability to recognize crucial atoms, bonds, functional groups, scaffolds, and moieties of small molecules in predicting biological activities against HBV and HCV. The molecular insights from this study broaden our understanding of the structure relationship of small molecules in the inhibition of HBV and HCV. This research can contribute to the discovery and development of HBV and HCV drugs, which may soon offer new therapeutic options that will benefit patients with HBV, HCV, or HBV/HCV co-infection.

### Supplementary Information

Supplementary Information 1.

Supplementary Information 2.

## Data Availability

All data, trained models, and source codes of HBCVTr, including those for training and predicting biological activity against HBV and HCV using SMILES, are available at https://github.com/imeewan/HBCVTr/.

## Abbreviations

HBV: Hepatitis B virus
HCV: Hepatitis C virus
SBDD: Structure-based drug design
LBDD: Ligand-based drug design
SMILES: Simplified molecular-input line-entry system
BART: Bidirectional and auto-regressive transformer
dsDNA: Double-stranded DNA
+ssRNA: Positive-sense single-stranded RNA
HBcAg: Hepatitis B core antigen
DAAs: Direct-acting antivirals
RdRp: RNA-dependent RNA polymerase
QSAR: Multi-target quantitative structure–activity relationship
QSBER: Multi-tasking quantitative structure–biological effect relationships
ML: Machine learning
DL: Deep learning
DNNs: Deep neural networks
ReLU: Rectified linear unit
LR: Linear regression
k-NN: K-nearest neighbors
SVR: Support vector regression
RF: Random forests
DT: Decision trees
GBM: Gradient boosted machines
RR: Ridge regression
Adaboost: Adaptive boosting
MSE: Mean square error
MAE: Mean absolute error
RMSE: Root mean square error
UPGMA: Unweighted pair group method with arithmetic mean
PCC: Pearson correlation coefficient
Spearman: Spearman rank correlation
RMSD: Root-mean-square deviation
PK: Pharmacokinetics
PD: Pharmacodynamics
PAINS: Pan assay interference compound
