# Supplementary material for: HBCVTr: an end-to-end transformer with a deep neural network hybrid model for anti-HBV and HCV activity predictor from SMILES
Source: Sci Rep. 2024 Apr 22;14:9262. doi: 10.1038/s41598-024-59933-4 (PMC11035669; doi:10.1038/s41598-024-59933-4)
Supplement: Supplementary file 1 — Supplementary Information 1. [file 41598_2024_59933_MOESM1_ESM.docx]

**Supplementary Data for HBCVTr: An end-to-end transformer with a deep neural network hybrid model for anti-HBV and HCV activity predictor from SMILES**

Ittipat Meewan^1*^, Jiraporn Panmanee^2^, Nopphon Petchyam^1^, Pichaya Lertvilai^3^

^1^Center for Advanced Therapeutics,  Institute of Molecular Biosciences, Mahidol University, Nakhon Pathom 73170, Thailand

^2^Research Center for Neuroscience, Institute of Molecular Biosciences, Mahidol University, Nakhon Pathom 73170, Thailand

^3^Scripps Institution of Oceanography, University of California San Diego, La Jolla, CA 92037, USA

**Supplementary Figures**
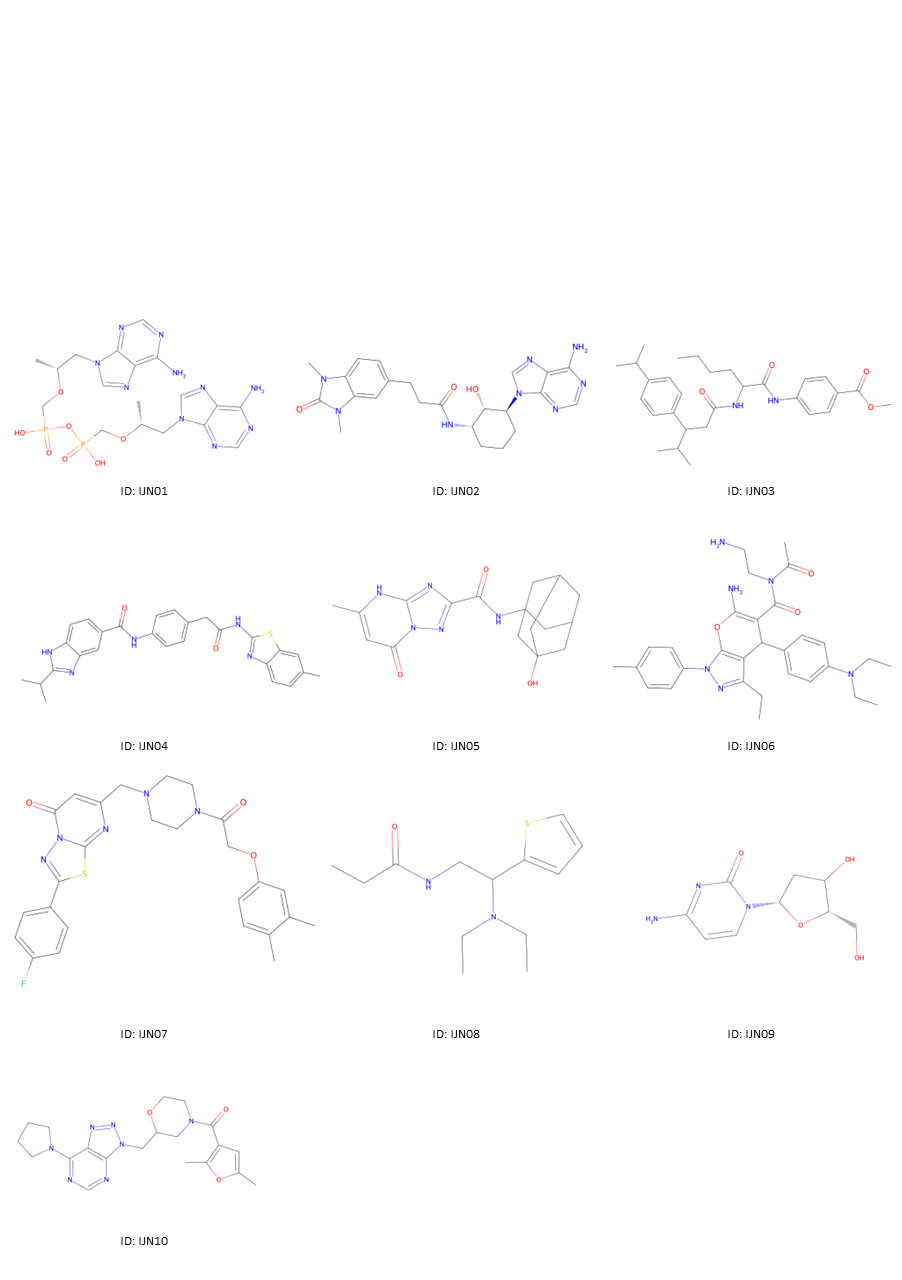


**Figure S1** The chemical structures of IJN01 to IJN10 representatives and their predicted biological activities against HBV.

**
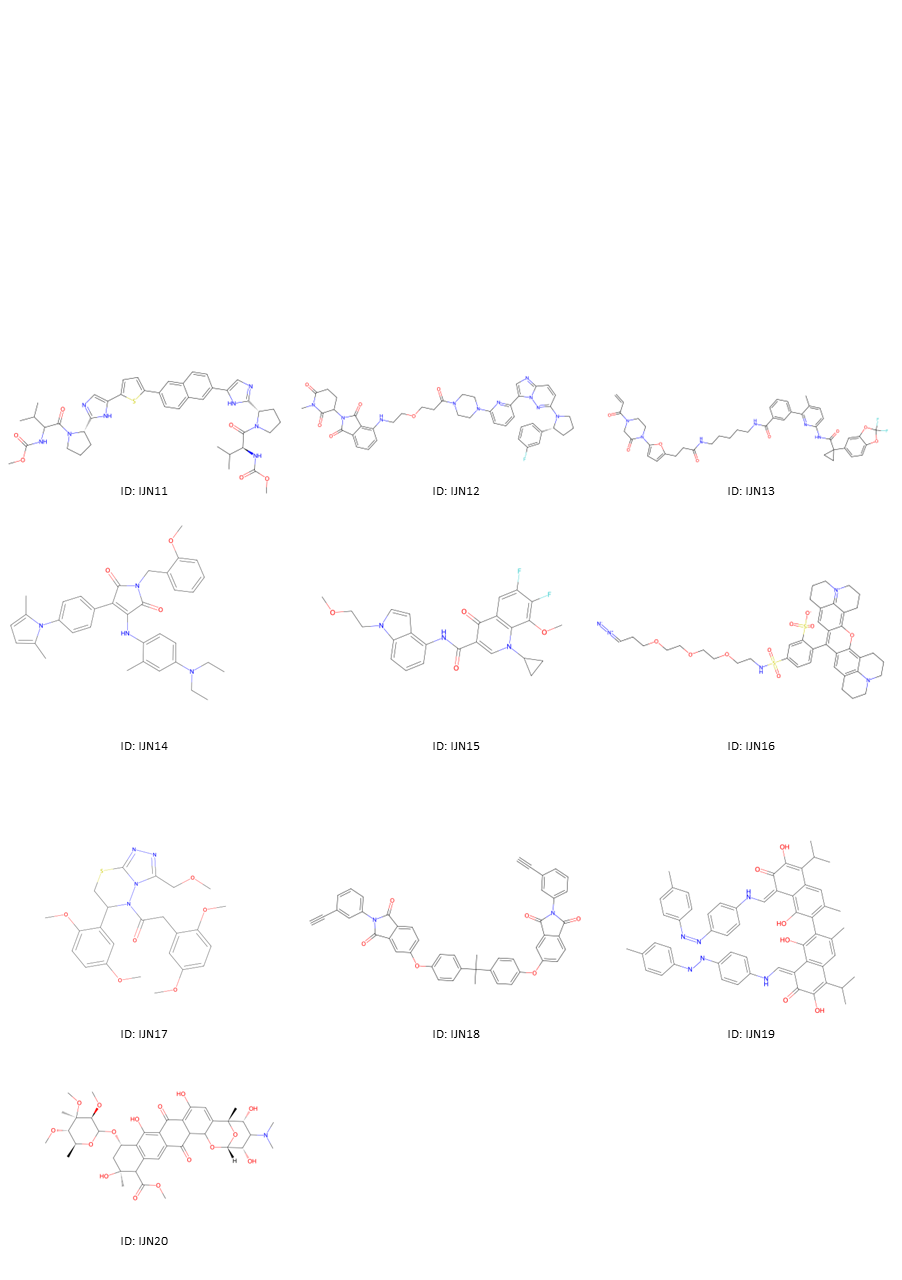
**

**Figure S2** The chemical structures of IJN11 to IJN12 representatives and their predicted biological activities against HCV.

**
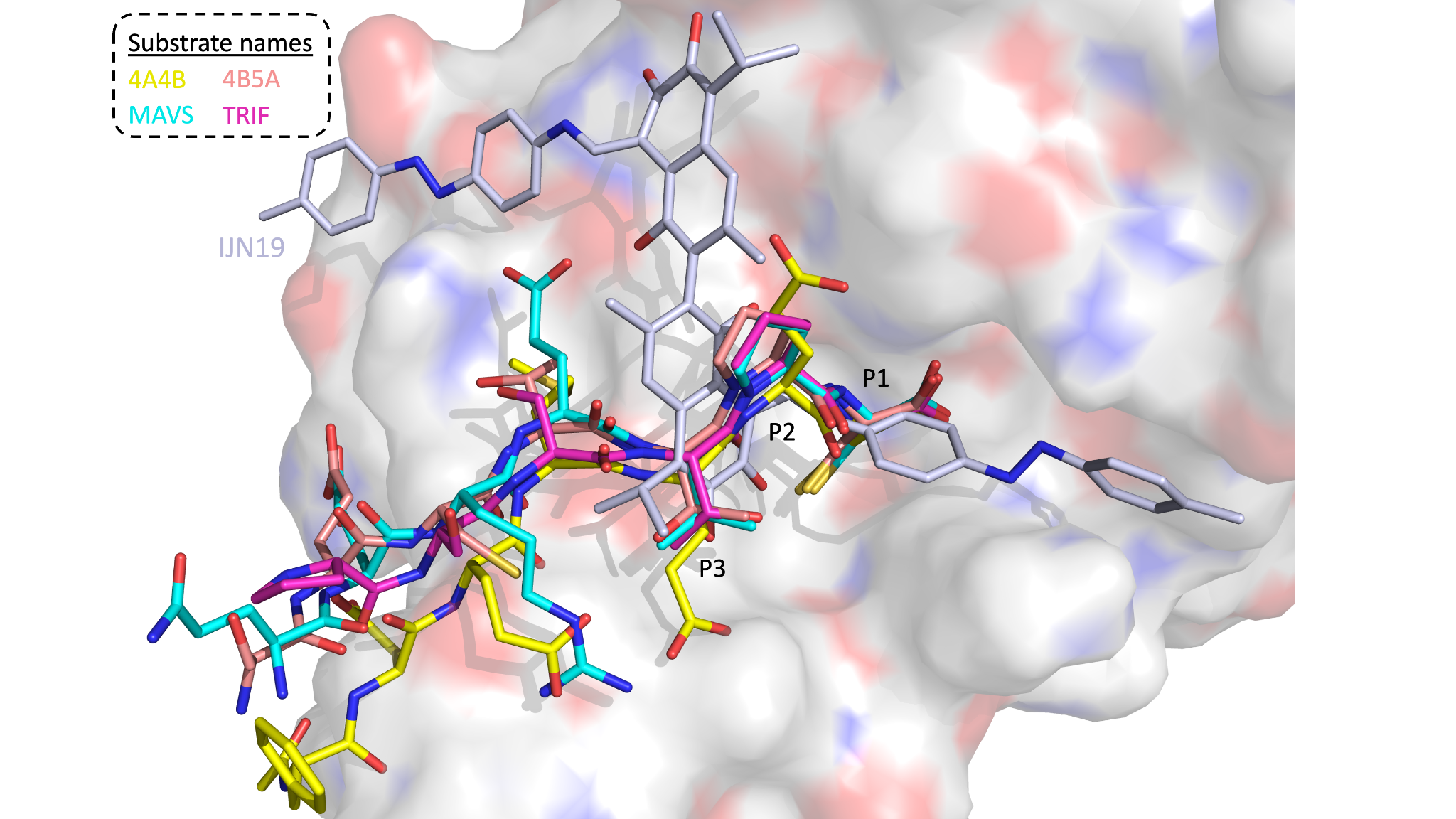
**

**Figure S3** Binding pose of IJN19 in comparison with peptide substrates in HCV NS3/4A protease. The docking site of IJN19 overlaps with the P1 to P3 positions of the peptide substrates, in which their 3D shapes are similar in the binding pocket. The peptide substrates include those from the host (TRIF shown in magenta from PDB code 3RC4, and MAVS shown in blue from PDB code 3RC5), and from the HCV (4A4B shown in yellow from PDB code 3M5M, and 4B5A shown in salmon from PDB code 3M5N). HCV NS3/4A protease is shown as a gray surface, and IJN19 as a gray stick.

**Supplementary Tables**

**Table S1.** Learnable weight and hyperparameters search

| Parameters | Range of parameters |
| --- | --- |
| Total encoder dimension (d_model) | [512, 1024] |
| Number of attention heads | [8, 16, 32, 64] |
| Number of encoder layers | [4, 6, 8, 12 ,16] |
| Number of feed forward dimension | [512, 1024, 2048, 4096] |
| Learning rate | [0.001, 0.00001, 0.0000001] |
| Weight decay | [0.0001, 0.001, 0.1] |
| Dropout rate | [0, 0.01, 0.025, 0.05, 0.1, 0.25] |
| Regression deep neural network size | [[1024, 640, 1], [2048, 640, 1]] |
| Number of epochs | [50, 200] |

**Table S2.** Parameters of reference machine learning models

| Model | Parameters |
| --- | --- |
| Logistic regression (LR) | {'copy_X': True, 'fit_intercept': True, 'n_jobs': None, 'normalize': 'deprecated', 'positive': False} |
| k-nearest neighbors (k-NN) | {'algorithm': 'auto', 'leaf_size': 30, 'metric': 'minkowski', 'metric_params': None, 'n_jobs': None, 'n_neighbors': 5, 'p': 2, 'weights': 'uniform'} |
| Support vector regression (SVR) | {'C': 1.0, 'cache_size': 200, 'coef0': 0.0, 'degree': 3, 'epsilon': 0.1, 'gamma': 'scale', 'kernel': 'rbf', 'max_iter': -1, 'shrinking': True, 'tol': 0.001, 'verbose': False} |
| Random forest (RF) | {'bootstrap': True, 'ccp_alpha': 0.0, 'criterion': 'squared_error', 'max_depth': None, 'max_features': 'auto', 'max_leaf_nodes': None, 'max_samples': None, 'min_impurity_decrease': 0.0, 'min_samples_leaf': 1, 'min_samples_split': 2, 'min_weight_fraction_leaf': 0.0, 'n_estimators': 100, 'n_jobs': None, 'oob_score': False, 'random_state': None, 'verbose': 0, 'warm_start': False} |
| Decision tree (DT) | {'ccp_alpha': 0.0, 'criterion': 'squared_error', 'max_depth': None, 'max_features': None, 'max_leaf_nodes': None, 'min_impurity_decrease': 0.0, 'min_samples_leaf': 1, 'min_samples_split': 2, 'min_weight_fraction_leaf': 0.0, 'random_state': None, 'splitter': 'best'} |
| XGBoost (XGB) | {'objective': 'reg:squarederror', 'base_score': None, 'booster': None, 'callbacks': None, 'colsample_bylevel': None, 'colsample_bynode': None, 'colsample_bytree': None, 'early_stopping_rounds': None, 'enable_categorical': False, 'eval_metric': None, 'feature_types': None, 'gamma': None, 'gpu_id': None, 'grow_policy': None, 'importance_type': None, 'interaction_constraints': None, 'learning_rate': None, 'max_bin': None, 'max_cat_threshold': None, 'max_cat_to_onehot': None, 'max_delta_step': None, 'max_depth': None, 'max_leaves': None, 'min_child_weight': None, 'missing': nan, 'monotone_constraints': None, 'n_estimators': 100, 'n_jobs': None, 'num_parallel_tree': None, 'predictor': None, 'random_state': None, 'reg_alpha': None, 'reg_lambda': None, 'sampling_method': None, 'scale_pos_weight': None, 'subsample': None, 'tree_method': None, 'validate_parameters': None, 'verbosity': None} |
| Gradient boosting (GB) | {'alpha': 0.9, 'ccp_alpha': 0.0, 'criterion': 'friedman_mse', 'init': None, 'learning_rate': 0.1, 'loss': 'squared_error', 'max_depth': 3, 'max_features': None, 'max_leaf_nodes': None, 'min_impurity_decrease': 0.0, 'min_samples_leaf': 1, 'min_samples_split': 2, 'min_weight_fraction_leaf': 0.0, 'n_estimators': 100, 'n_iter_no_change': None, 'random_state': None, 'subsample': 1.0, 'tol': 0.0001, 'validation_fraction': 0.1, 'verbose': 0, 'warm_start': False} |
| Ridge | {'alpha': 1.0, 'copy_X': True, 'fit_intercept': True, 'max_iter': None, 'normalize': 'deprecated', 'positive': False, 'random_state': None, 'solver': 'auto', 'tol': 0.001} |
| Adaptive boosting | {'base_estimator': None, 'learning_rate': 1.0, 'loss': 'linear', 'n_estimators': 50, 'random_state': None} |
| Mol2vec^1^ | {‘radius’: 1, ‘dimension’: 300} |

**Reference:**

1. Jaeger, S., Fulle, S. & Turk, S. Mol2vec: Unsupervised Machine Learning Approach with Chemical Intuition. *J. Chem. Inf. Model.* **58**, 27–35 (2018).
